# Multidrug Antimicrobial Resistance and Molecular Detection of *mcr-1* Gene in *Salmonella* Species Isolated from Chicken

**DOI:** 10.3390/ani11010206

**Published:** 2021-01-15

**Authors:** Md Bashir Uddin, S.M. Bayejed Hossain, Mahmudul Hasan, Mohammad Nurul Alam, Mita Debnath, Ruhena Begum, Sawrab Roy, Ahmed Harun-Al-Rashid, Md. Shahidur Rahman Chowdhury, Md. Mahfujur Rahman, Md. Mukter Hossain, Fazle Elahi, Mohammed Yousuf Elahi Chowdhury, Josef D. Järhult, Mohamed E. El Zowalaty, Syed Sayeem Uddin Ahmed

**Affiliations:** 1 Department of Medicine, Sylhet Agricultural University, Sylhet 3100, Bangladesh; dr.bayejed@gmail.com (S.M.B.H.); dr.nurul_alam@ymail.com (M.N.A.); shahidur.vetmed@sau.ac.bd (M.S.R.C.); mahfuj.vetmed@sau.ac.bd (M.M.R.); mukter.vetmed@sau.ac.bd (M.M.H.); 2 Department of Pharmaceuticals and Industrial Biotechnology, Sylhet Agricultural University, Sylhet 3100, Bangladesh; mhasan.pib@sau.ac.bd; 3 Kazi Farms Poultry Laboratory, Gazipur 1700, Bangladesh; debnath.mita@gmail.com; 4 Bangladesh Livestock Research Institute, Dhaka 1341, Bangladesh; dr.ruhenabegum@gmail.com; 5 Department of Microbiology and Immunology, Sylhet Agricultural University, Sylhet 3100, Bangladesh; sawrab_sau@yahoo.com; 6 Department of Aquatic Resource Management, Sylhet Agricultural University, Sylhet 3100, Bangladesh; russel.sau@gmail.com; 7 Department of Food Science and Biotechnology, Kangwon National University, Chuncheon 200-701, Korea; elahidr@gmail.com; 8 Department of Medicine and Surgery, Chattogram Veterinary and Animal Sciences University, Chattogram 4225, Bangladesh; dalim2000@gmail.com; 9 Zoonosis Science Center, Department of Medical Sciences, Uppsala University, SE 751 85 Uppsala, Sweden; josef.jarhult@medsci.uu.se; 10 Department of Clinical Sciences, College of Medicine, University of Sharjah, Sharjah 27272, UAE; 11 Zoonosis Science Center, Department of Medical Biochemistry and Microbiology, Uppsala University, SE 75 123 Uppsala, Sweden; 12 Department of Epidemiology and Public Health, Sylhet Agricultural University, Sylhet 3100, Bangladesh

**Keywords:** antimicrobial resistance, *Enterobacteriaceae*, colistin, *mcr-1* gene, *Salmonella enterica*, foodborne, poultry, Bangladesh, chicken, phosphoethanolamine, LptA

## Abstract

**Simple Summary:**

Colistin is a widely used antibiotic against infections caused by extensively drug-resistant Gram-negative bacteria. It is critical to track and monitor the presence of *mcr*-like genes and colistin resistance to protect a last resort treatment against highly antibiotic-resistant bacteria. In the present study, the colistin resistance gene *mcr-1* was investigated, and its in-silico functional analysis in *Salmonella* isolates was determined. Out of 100 chicken samples (liver and intestine), 82 *Salmonella* spp. were isolated and characterized. Antimicrobial sensitivity was determined using different antimicrobial agents. The isolates were characterized using PCR targeting genus-specific *invA* and *mcr-1* genes followed by in-silico functional analysis. The majority of isolates (92.68%) was found highly resistant to colistin, which demonstrated the occurrence of the colistin resistance *mcr-1* gene in *Salmonella* isolates of chicken origin in Bangladesh. The study also showed the in-silico functional analysis and phylogenetic relationship of the colistin resistance *mcr-1* gene among *Salmonella* isolates. The findings of the present study highlight the increasing issue of transferable colistin resistance and call for immediate action and measures to review the imprudent use of colistin in poultry production systems in Bangladesh.

**Abstract:**

Colistin (polymyxin E) is widely used in animal and human medicine and is increasingly used as one of the last-resort antibiotics against Gram-negative bacilli. Due to the increased use of colistin in treating infections caused by multidrug-resistant Gram-negative bacteria, resistance to this antibiotic ought to be monitored. The study was undertaken to elucidate the molecular mechanisms, genetic relationships and phenotype correlations of colistin-resistant isolates. Here, we report the detection of the *mcr-1* gene in chicken-associated *Salmonella* isolates in Bangladesh and its in-silico functional analysis. Out of 100 samples, 82 *Salmonella* spp. were isolated from chicken specimens (liver, intestine). Phenotypic disc diffusion and minimum inhibitory concentration (MIC) assay using different antimicrobial agents were performed. *Salmonella* isolates were characterized using PCR methods targeting genus-specific *invA* and *mcr-1* genes with validation for the functional analysis. The majority of the tested *Salmonella* isolates were found resistant to colistin (92.68%), ciprofloxacin (73.17%), tigecycline (62.20%) and trimethoprim/sulfamethoxazole (60.98%). When screened using PCR, five out of ten *Salmonella* isolates were found to carry the *mcr-1* gene. One isolate was confirmed for *Salmonella enterica* subsp. *enterica* serovar Enteritidis, and other four isolates were confirmed for *Salmonella enterica* subsp. *enterica* serovar Typhimurium. Sequencing and phylogenetic analysis revealed a divergent evolutionary relationship between the catalytic domain of *Neisseria meningitidis* lipooligosaccharide phosphoethanolamine transferase A (LptA) and MCR proteins, rendering them resistant to colistin. Three-dimensional homology structural analysis of MCR-1 proteins and molecular docking interactions suggested that MCR-1 and LptA share a similar substrate binding cavity, which could be validated for the functional analysis. The comprehensive molecular and in-silico analyses of the colistin resistance *mcr-1* gene of *Salmonella* spp. of chicken origin in the present study highlight the importance of continued monitoring and surveillance for antimicrobial resistance among pathogens in food chain animals.

## 1. Introduction

In spite of the significant improvements in the poultry sector in Bangladesh [1], there is a potential risk of infectious diseases due to associated bacterial pathogens, which can result in huge economic losses [2]. Infections due to *Salmonella* spp. are the most commonly reported bacterial diseases in poultry and may cause foodborne illnesses in human. Salmonellosis remains a persistent threat to both human and animal health [2,3]. Although vaccination and good hygienic practices are among the most effective measures to prevent salmonellosis [4], antibiotics are extensively used either as growth promoters [5,6], prophylactic agents, or therapeutics in the poultry industry in Bangladesh [7]. The widespread misuse and overuse of antimicrobial agents in poultry settings contribute to the emergence and the development of antimicrobial resistance in livestock pathogens such as *Salmonella* [8].

Antimicrobial-resistant *Salmonella* in poultry is a potential risk and a common vector for the dissemination of antimicrobial resistance to humans [8,9,10]. Standard microbiological and serological methods are usually employed for isolation and identification of *Salmonella* species. However, the invasion gene *invA*, commonly involved in bacterial virulence, is also routinely used for the accurate detection of *Salmonella* spp. in clinical samples [11]. The nucleotide sequences of the *invA* gene are distinctive to the genus *Salmonella*, and the amplification of the *invA* gene by the polymerase chain reaction (PCR) is a suitable diagnostic method of detection of *Salmonella* due to its reliability, sensitivity, specificity and detection speed [12]. Of note, poultry is a major source of *Salmonella* infections that cause mild to severe illnesses in humans. Therefore, the detection of *Salmonella* species in the poultry production food chain, particularly at the farm level, is of great concern. Furthermore, the escalating antimicrobial resistance of some *Salmonella* serotypes to multiple antibiotics [8] has led to the study of antimicrobial susceptibility profiles and the underlying resistance genetic determinants of priority [13].

Recently, the colistin resistance *mcr-1* gene was firstly reported from animals and human in China [14]. Subsequently, *mcr-1* positive *Enterobacteriaceae* was reported in animals, humans, food, and the environment in more than 25 countries across 4 continents, and the majority of studies reported *mcr-1* positive *Escherichia* (*E.*) *coli* [14,15,16]. In comparison to *E. coli*, very few studies in Europe reported *mcr-1* detection in *Salmonella* in poultry [17]. However, there is no data from Bangladesh in the literature on the molecular characterization and *mcr-1* gene detection in *Salmonella* in poultry. Therefore, the present study was conducted to detect the colistin resistance *mcr-1* gene in *Salmonella* isolates of chicken origin and to study the associated antimicrobial resistance patterns. The study was also conducted to elucidate the molecular mechanisms, genetic relationships and phenotype correlations of colistin-resistant *Salmonella* isolates. Phylogenetic relationships between the tested local *Salmonella* isolates and other published data from different parts of the world were also analyzed. Additionally, molecular docking of the phosphatidylethanolamine substrate with MCR-1 and LptA were investigated.

## 2. Materials and Methods

### 2.1. Ethical Standards

The experimental protocol was reviewed and approved by the Animal Experimentation Ethics Committee of Sylhet Agricultural University (SAU) (approval number #AUP2018004).

### 2.2. Study Area and Sampling

**Figure 1:**
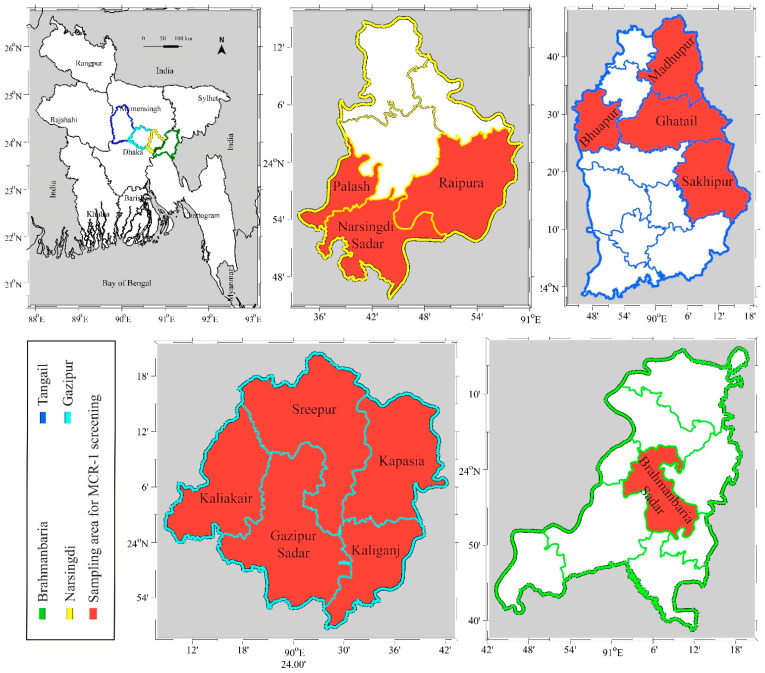
Geographical map of Bangladesh showing the locations of the sampling areas in selected districts in the present study. Areas where the colistin resistance gene *mcr-1* was screened are highlighted in red.

The study was conducted from January 2019 to June 2019 in widely occupied poultry zones of Gazipur, Narsingdi, Tangail and Brahmanbaria in Bangladesh (Figure 1).

Selected dead and sick chicken (*Gallus gallus domesticus*) were randomly collected and transferred to the laboratory. Postmortem examinations were immediately conducted on receiving the birds, and the anamnesis and clinical information were collected by a local veterinarian in accordance with the standard guidelines. During postmortem examinations, liver and intestinal tissue samples were collected for further analysis. Blood samples were collected from sick live chicken and sera samples were prepared before the postmortem examination.

### 2.3. Isolation and Biochemical Identification of Bacterial Isolates

Before isolation of *Salmonella* spp., samples were initially screened using a rapid serum plate agglutination test (Innovative Diagnostics, Grabels, France) followed by clinical and postmortem examinations. Liver and intestinal samples from 100 *Salmonella* suspected infected chicken were subjected to pre-enrichment step by culturing in 225 mL buffered peptone water medium in a ratio of 1:10 and samples were incubated at 37 °C for 24 h. For *Salmonella* specific pre-enrichment, cultures were further transferred to modified semi-solid Rappaport-Vassiliadis (MSRV) agar (HiMedia Laboratories Pvt. Ltd., Mumbai, India) and tetrathionate (TT) broth (HiMedia Laboratories Pvt. Ltd., Mumbai, India) consecutively and were incubated at 42 °C for 24 h. Following enrichment, a loopful of enriched broth was initially streaked on *Salmonella–Shigella* (SS) agar, and pure colonies (single pinkish with black center) were streaked on xylose–lysine–deoxycholate (XLD) agar (HiMedia Laboratories Pvt. Ltd, Mumbai, India) and plates were incubated at 37 °C for 24 h. Colonies were identified as *Salmonella* based on morphological and biochemical properties using Gram’s stain, catalase test and indole test as was previously described [18]. For further confirmation, 4–6 colonies from each sample were tested biochemically by dilution streaking and stabbed onto triple sugar iron (TSI) agar (Merck, Germany) and tubes were incubated at 37 °C for 16–24 h [19].

### 2.4. Antimicrobial Susceptibility Testing

Antibiogram profiles were obtained for *Salmonella* isolates using 19 antimicrobial agents by the Kirby Bauer’s disk diffusion method as previously described [20]. The results were interpreted by measuring the zones of inhibition (in mm), and results were reported as sensitive, intermediate or resistant according to CLSI guidelines [21]. The minimum inhibitory concentrations (MICs) of the tested antimicrobials were determined as described by global CLSI based and natural resistance [21,22]. MIC was performed using a VITEK 2 compact AST card N280. *E. coli* (ATCC 25922) was used as the quality-control strain. The susceptibility breakpoints of the tested antimicrobials were interpreted based on CLSI-EUCAST plus natural resistance guidelines [21,22]. Isolates that were found resistant to at least 3 different classes of antibiotics were considered multidrug-resistant (MDR) isolates [23].

### 2.5. Bacterial DNA Extraction

Ten isolates out of the 82 phenotypically identified *Salmonella* cultures were randomly selected and were subjected to further testing using *Salmonella* spp. detection kit (AddBio Inc. Ltd., Daejeon, South Korea) using PCR molecular methods for the detection of the *invA* gene. Bacterial DNA was extracted from the 10 isolates using the boiling method as previously described [24]. Briefly, a loopful of an overnight culture suspension was heated at 100 °C for 8–10 min in a heating block and then immediately cooled on ice for 5 min. Cellular debris was pelleted by centrifugation at 13,000 rpm for 1 min, and the remaining supernatant was used as the DNA template in PCR molecular tests.

### 2.6. Polymerase Chain Reaction (PCR) and Gel Electrophoresis

Extracted DNA was subjected to PCR for the initial confirmation of *Salmonella* isolates using specific primers targeting *invA* gene (forward 3′ TAATGCCAGACGAAAGAGCGT 5′ and reverse 3′ GATA TTGGTGTTTATGGGGTCGTT 5′) as previously described [25] using a *Salmonella* spp. detection kit (AddBio Inc. Ltd., Daejeon, South Korea) according to the manufacturer guidelines. Lambda primers (forward sequence 3′ CAGATCTCCAGCACGGAACTATTGAGTACGAACG 5′ and reverse sequence 5′ GCATAAAATGCGGGGATTCACTGGCTGC 3′) were used as internal controls, and an expected amplicon size of 100 bp indicated positive *Salmonella* spp. (AddBio Inc. Ltd., Daejeon, South Korea). The reaction volume was 20 µL and consisted of 10 µL of 2X master mix with uracil–DNA glycosylase (UDG), 5 µL of primer mix and 5 µL of DNA sample. Forward and reverse primers were used at 5 pmole each per reaction. The PCR was performed using a DLAB TC100-G machine (DLAB Scientific Co., Ltd., Beijing, China), and reaction conditions were as follows: UDG reaction for 3 min at 50 °C, initial denaturation for 10 min at 95 °C, 35 cycles denaturation for 30 s at 95 °C, primer annealing and extension for 45 s at 68 °C followed by a final elongation step for 5 min at 72 °C.

For the detection of *mcr-1* colistin resistance genes, DNA extracts of the 10 *Salmonella* isolates were subjected to PCR reaction using designed primers (NeoProbe, Daejeon, South Korea), as shown in Table 1, which cover the reading frame of *mcr-1* gene with the allocated two amplicons (1197 bp and 799 bp). For the primer design, *mcr-1* gene sequences were retrieved from NCBI and sequences were aligned using the BioEdit version 7.2 (Ibis Biosciences, http://www.mbio.ncsu.edu/bioedit/bioedit.html). Two primers were designed using the Primer3 program available at NCBI, and these two primers yielded two amplicons (1197 bp and 799 bp), which covered the coding sequence of the full-length *mcr-1* gene (1626 bp). The reaction volume was 20 µL and consisted of 10 µL of 2X master mix, 5 µL of primer mix and 5 µL of DNA sample. Forward and reverse primers were used at 5 pmole each per reaction. The PCR was performed using a DLAB TC100-G machine (DLAB Scientific Co., Ltd., Beijing, China), and reaction conditions were as follows: initial denaturation for 10 min at 95 °C, 40 cycles denaturation for 30 s at 95 °C, primer annealing and extension for 45 s at 68 °C followed by a final elongation step for 5 min at 72 °C. The amplified products were visualized by gel electrophoresis using 1.5% agarose gel and viewed under UV transillumination in a gel documentation system (Bio-Rad, Hercules, CA, USA).

### 2.7. Sequencing, Multiple Sequence Alignment and Phylogenetic Analysis

Amplicons of *mcr-1* gene were purified using a DNA purification kit (AddBio Inc. Ltd., Daejeon, South Korea) and were subjected to Sanger sequencing (SolGent Co., Ltd., Daejeon, South Korea). Sequences were checked using BLAST search tool and annotated to GenBank. In case of *invA* gene, five representative *invA* gene amplicons were sequenced from 10 positive *invA* gene isolates to confirm the results and the identity of *invA* gene PCR amplicons using the BLAST search tool (Supplementary File S1).

The BLASTp search tool (https://blast.ncbi.nlm.nih.gov/Blast.cgi) was used to retrieve the homologous sequences of the MCR-1 and MCR-1 like proteins containing LptA (formerly named EptA) and others from the NCBI database using five SAUVM MCR-1 proteins translated from the *mcr-1* genes of *Salmonella* spp. (Supplementary Files S2 and S3). Multiple sequence alignment of *Salmonella* SAUVM MCR-1 proteins and retrieved *MCR-1* of *Salmonella* species was performed using T-Coffee default parameters [26]. The maximum likelihood method of MEGA X [27] was used to construct a phylogenetic tree using aligned sequences of MCR-1 from ClustalW [28]. Results were validated using 500 bootstrap replicates.

### 2.8. Transmembrane Topology Analysis, Structural Modelling, Refinement and Validation

To predict the transmembrane helices of MCR-1 proteins, the TMHMM server v.2.0 (Department of Health Technology, Lyngby, Denmark; http://www.cbs.dtu.dk/services/TMHMM/) was used with standard parameters. The topology was given as the position of the transmembrane helices differentiated by “I” and “o” when the loop is on the inside and outside, respectively [29]. Three dimensional (3D) modeling of *Salmonella* SAUVM MCR-1 proteins was performed by I-TASSER, which functions by identifying structure templates from the Protein Data Bank (PDB) library. The confidence of each model was quantitatively measured by C-score [30]. To enhance the accuracy of the predicted structures, refinement was performed using ModRefiner [31] followed by the FG-MD refinement server [32]. Finally, the refined structures were also validated using the Verified 3D, ERRAT and Ramachandran Plot Assessment server (RAMPAGE) [33].

### 2.9. Molecular Docking of Phosphatidylethanolamine Substrate with mcr-1 and LptA

The chemical structure of phosphatidylethanolamine (ZINC identification number (ID): ZINC32837869) was obtained from the ZINC database [34], while the 3D structure of LptA (PDB ID: 5FGN; *Neisseria meningitidis*), the best template of MCR-1 of *Salmonella* SAUVM isolates was retrieved from the RCSB Protein Data Bank server [35]. Binding interactions of the phosphatidylethanolamine to the MCR-1 LptA were investigated by molecular docking using the Autodock Vina algorithm in PyRx software [36]. OpenBabel (version 2.3.1) was used to convert the output PDBQT files into PDB format. PyMol and Discovery Studio software were used to optimize and visualize the protein structures and ligand binding interaction patterns [37].

## 3. Results

### 3.1. Confirmation of Salmonella spp.

A total of 82 *Salmonella* spp. were isolated from 100 samples based on their morphological and biochemical properties. Out of 82 isolates, 10 *Salmonella* isolates were randomly selected for further confirmation by PCR molecular methods for the presence of the *invA* virulence gene. It was found that all the 10 *Salmonella* isolates tested were *invA* gene positive. One isolate was subjected to 16S rRNA. The obtained sequences of five *mcr-1* genes were checked using the BLAST search tool (https://blast.ncbi.nlm.nih.gov/Blast.cgi) and annotated to GenBank. Among the five *mcr-1* positive *Salmonella* isolates, one isolate (GenBank accession numbers of the forward and reverse 16S rRNA partial sequences are MW425840 and MW425841, respectively) was confirmed as *Salmonella enterica* subsp. *enterica* serovar Enteritidis using 16S rRNA analysis, and four isolates were confirmed as *Salmonella enterica* subsp. *enterica* serovar Typhimurium based on *mcr-1* gene similarity to other published *mcr-1* sequences of *Salmonella enterica* serovar Typhimurium.

### 3.2. Antimicrobial Susceptibility Testing

All *Salmonella* isolates (*n* = 82) were subjected to antibiotic resistance testing using 19 antimicrobial agents (Table 2). In general, a considerable percentage of resistance was observed among the tested *Salmonella* isolates using the disk diffusion test according to CLSI guidelines [21]. Specifically, high percentage of resistance were found against colistin (92.68%), ciprofloxacin (73.17%), tigecycline (62.20%), trimethoprim/sulfamethoxazole (60.98%), amoxicillin/clavulanate (41.46%) and ceftriaxone (40.24%). The susceptibility rates of the tested *Salmonella* isolates were for ampicillin (85.37%), piperacillin/tazobactam (84.15%), nitrofurantoin (73.17%), imipenem (68.29%) and amikacin (64.63). The multidrug resistance (MDR) patterns were also evaluated by MIC among *mcr-1* positive *Salmonella* spp. isolates.

All the tested *mcr-1* positive *Salmonella* isolates (*n* = 5) showed a MDR phenotype, where all five isolates were resistant to at least three different classes of antimicrobial agents (at least colistin, ciprofloxacin and trimethoprim/sulfamethoxazole) (Table 3). As shown in Table 3, three out of five tested isolates (60%) were resistant to six antibiotics, and two out of five isolates (40%) were resistant to seven antibiotics.

### 3.3. Detection of Salmonella Isolates Using PCR and Sequencing of mcr-1 Genes

**Figure 2:**
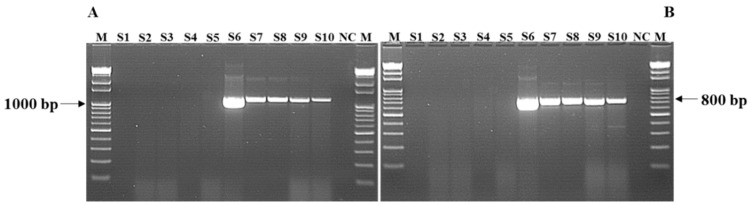
PCR amplification of antimicrobial resistance *mcr-1* gene of *Salmonella* isolates. In *Salmonella* (S6 to S10) isolates, fragments of (**A**) 1197 bp and (**B**) 799 bp were detected. Lane M: DNA ladder. Lane NC: negative control. Lanes S1 to S5 represents *mcr-1* negative isolates while lanes S6 to S10 represents *mcr-1* positive *Salmonella* isolates SAUVM S6 to SAUVM S10.

Five out of 10 randomly selected *Salmonella* isolates obtained from chickens were found positive for *mcr-1* using primer-specific PCR (Figure 2). The five *mcr-1* carrying *Salmonella* spp. strains isolated from chicken in the present study were deposited to NCBI under bioproject number PRJNA687398 with accession numbers SAMN17142409, SAMN17142425, SAMN17142450, SAMN17142964, SAMN17145260 for SAUVM S6, SAUVM S7, SAUVM S8, SAUVM S9 and SAUVM S10, respectively.

The sequences of the *mcr-1* genes of *Salmonella* spp. were deposited into the GenBank under accession numbers MN873694, MN873695, MN873696, MN873697 and MN873698 for SAUVM S6, SAUVM S7, SAUVM S8, SAUVM S9 and SAUVM 10, respectively.

DNA sequencing of the *mcr-1* amplicons confirmed them as *mcr-1*, and the obtained sequences showed 100% similarity in four isolates (Supplementary File S4), while one isolate SAUVM S9 (accession number MN873697) contained a new allele of the MCR-1 family (mcr-1.23 allele) than others retrieved from the NCBI database.

### 3.4. Sequence Acquisition, Multiple Sequence Alignment and Phylogenetic Analysis

**Figure 3:**
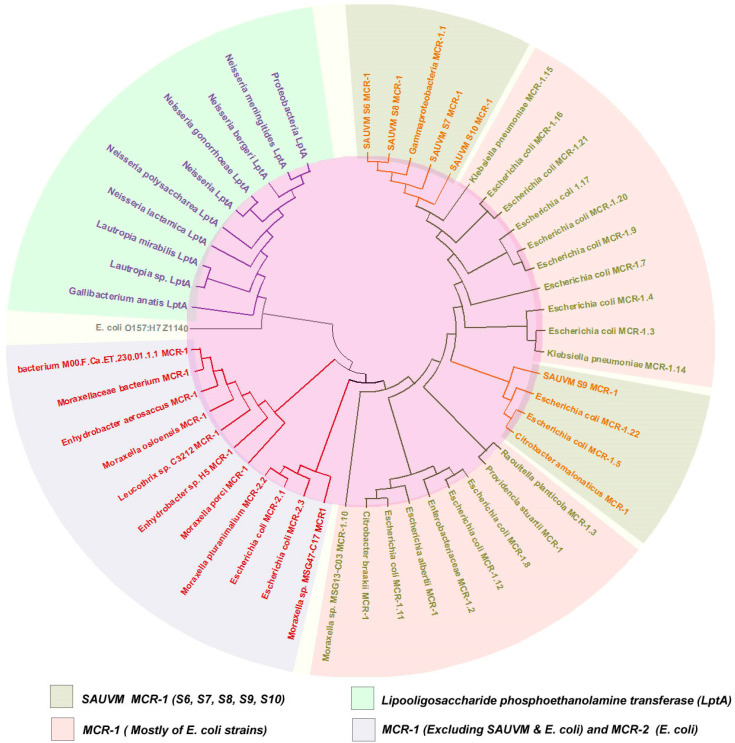
Phylogeny analysis showing ancestral origin and diversification of MCR-1 and MCR-1 like proteins. The *BLAST* search tool (https://blast.ncbi.nlm.nih.gov/Blast.cgi) was used to retrieve the homologous sequences of the MCR-1 and MCR-1 like proteins from the NCBI database using amino acid sequences of five SAUVM MCR-1 proteins. Sequences were categorized into MCR-1 and MCR-1 like proteins of *Salmonella, E. coli*, strains containing LptA (formerly named EptA) and others. The maximum likelihood method of MEGA X was used to construct a phylogenetic tree using aligned sequences of MCR-1 from CLUSTALW.

In order to analyze sequence similarities, phylogeny and structural insights of *mcr-1* gene products, the respective translated MCR-1 proteins were subjected to different bioinformatics analyses. A total of 52 homologous sequences of the MCR-1 and MCR-1 like proteins were retrieved from the NCBI database, while 44 sequences were subjected to phylogenetic analysis including SAUVM MCR-1 proteins. Again, eight MCR-1 proteins of *Salmonella* spp. were aligned with SAUVM MCR-1 proteins for further analysis. The evolutionary relation inferred via phylogeny analysis is given in Figure 3. The phylogenetic analysis revealed that all of the MCR-1 and MCR-1 like proteins were distinctly categorized into two major groups, namely chromosomally-encoded LptA and plasmid encoded MCR types, indicating a divergent evolutionary relation between the LptA and MCR proteins.

### 3.5. Transmembrane Topology Analysis, Structural Modelling, Refinement and Validation

**Figure 4:**
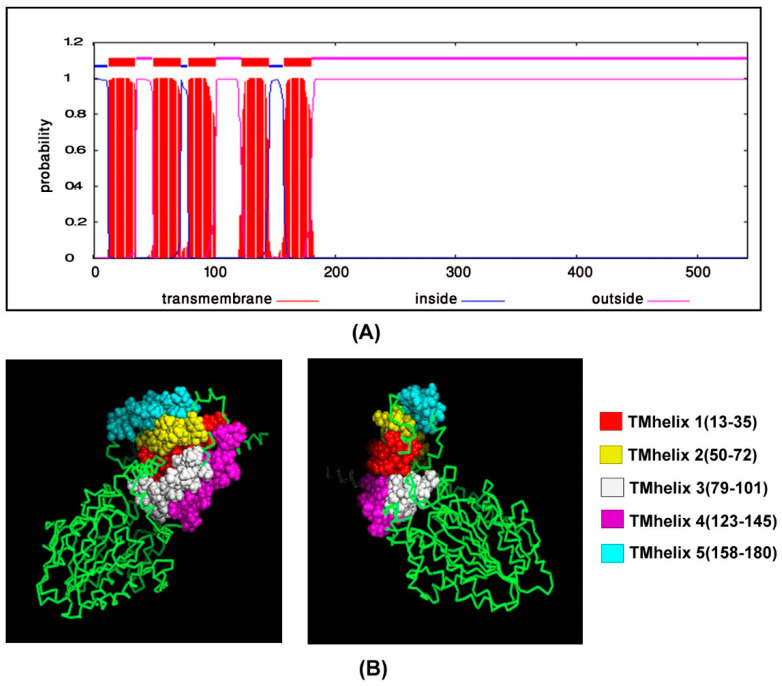
Transmembrane topology prediction of *Salmonella* SAUVM S6 MCR-1 protein. TMHMM server v.2.0 (http://www.cbs.dtu.dk/services/TMHMM/) was used to predict the (**A**) transmembrane helices of MCR-1 proteins and the image showed TMHMM posterior probabilities of SAUVM S6. (**B**) The topology was shown as the position of the transmembrane helices differentiated by “i” and “o” when the loop is on the inside and outside, respectively.

Prediction of transmembrane helices is significant in functional analysis of proteins. Therefore, the TMHMM server was used to predict transmembrane helices in *mcr-1* genes of colistin-resistant *Salmonella* isolates in the present study. TMHMM predicted that there were five transmembrane domains in the *Salmonella* SAUVM MCR-1 proteins, namely TMhelix1 (13–35), TMhelix 2 (50–72), TMhelix 3 (79–101), TMhelix 4 (123–145) and TMhelix 5 (158–180), which spanned the inner membrane region (Figure 4).

**Figure 5:**
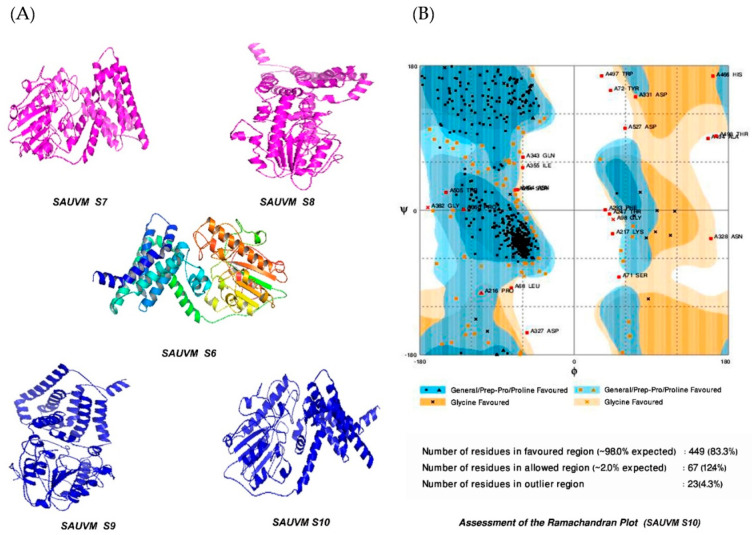
Modelled structures of *Salmonella* SAUVM MCR-1 proteins and validation. (**A**) Three dimensional (3D) modeling of *Salmonella* SAUVM MCR-1 proteins (SAUVM S6 to SAUVM S10) were performed using I-TASSER, which functions by identifying structure templates from the PDB library. The confidence of each model was quantitatively measured by C-score. From these models of MCR-1 proteins, the *Salmonella* SAUVM S10 model was randomly selected, (**B**) analyzed and structurally validated with the Ramachandran plot assessment server (RAMPAGE).

The structure of *Salmonella* SAUVM MCR-1 protein was modeled using the I-TASSER server, where EptA (PDB ID: 5FGN) of *Nisseria meningitidis* was used as the structural template. *Salmonella* SAUVM MCR-1 proteins showed 35.4% (35.6%) identity to EptA, and their modeled structure possessed a coverage score of 96% compared with that of EptA. Refinement was performed to enrich the quality of predicted structures beyond the accuracy. After refinement, Ramachandran plot analysis revealed that 83.3% of residues were in the favored, 12.4% of residues were in the allowed while only 4.3% of residues were in the outlier region (Figure 5). Moreover, ERRAT showed a 94.4% quality factor (Supplementary Figure S1A,B), and Verify3D suggested that 94.74% of the residues showed an average 3D-1D score of ≥0.2 (Supplementary Figure S2).

### 3.6. Molecular Docking of PE Substrate with MCR-1 and LptA

**Figure 6:**
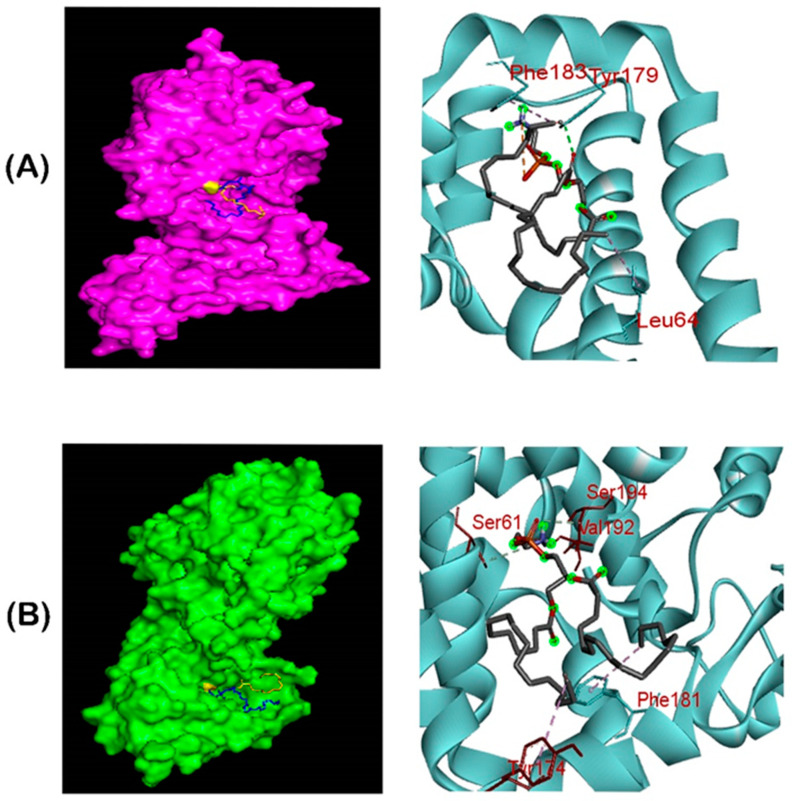
Ligand–binding interaction pattern of phosphatidylethanolamine substrate with colistin resistance MCR-1 and LptA. The modeled ribbon structure for PE substrate with MCR-1 protein. The ribbon structure was given via PyMol software. In both (**A**,**B**) cases, the substrates tend to bind in the groove of MCR-1 and LptA, mostly spanning the 175–195 region, in which Phe, Tyr and Ser residues were abundantly found in the substrate binding region for phosphatidylethanolamine interaction. (**B**) Ligand–binding interaction revealed that both MCR-1 and LptA proteins exhibited similar localization of phosphatidylethanolamine binding sites (SAUVM MCR-1: Leu 64, Tyr 179, Phe 183; LptA: Ser 61, Tyr 174, Phe 181, val 192, Ser 194).

The grid box was set to 82.0138 A° x 82.7041 A° x 82.471 A° (x, y and z) with 1 A° spacing between the grid points, while other parameters were left as default. Molecular docking of the PE substrate to SAUVM MCR-1 and LptA generated five binding conformations for each docking, but the binding pattern with the lowest energy was selected (PE and MCR-1: −3.4kcal/mol and PE and LptA: −3.6 kcal/mol). It was demonstrated that Leu 64, Tyr 179 and Phe 183 were the key interactive molecules in the PE binding cavity of SAUVM MCR-1, whereas Ser 61, Tyr 174, Phe 181, Val 192 and Ser 194 were the key interactive molecules for LptA (Figure 6).

## 4. Discussion

In the present study, out of 100 suspected clinical samples obtained from chicken in different poultry zones, 82 samples were confirmed as containing *Salmonella* spp. using conventional phenotypic methods and molecular methods of PCR and DNA sequencing of the *invA* gene. The finding of 82% of the tested samples in the present study containing *Salmonella* is similar to other studies that reported that apparently healthy commercial poultry farms and their surrounding environments may be a potential source of *Salmonella* spp. [38].

Molecular methods were used for identification of *Salmonella* isolates and the *mcr-1* gene using specific primers followed by confirmation using DNA sequencing and phylogenetic analysis. The pathogenesis of *Salmonella* is due to a combination of chromosomal and plasmid-mediated factors. A number of genes such as *invA*, *fimA*, *stn* and *spv* account for salmonellosis, of which the chromosomally located invasion (*invA)* gene codes for a protein in the inner membrane of the bacterium, which is necessary for invasion of the host epithelial cells [11,12]. In the present study, detection of the *invA* gene in 10 tested *Salmonella* isolates indicated their pathogenic nature. Antimicrobial resistance in livestock associated *Salmonella* spp., including fluoroquionolone and colistin resistant *Salmonella* spp., is an emerging global public health threat [4]. In the present study, antimicrobial susceptibility results showed that the majority of the tested *Salmonella* isolates were found resistant to colistin, ciprofloxacin and tigecycline. This high resistance rates in the present study reflects the widespread use of antibiotics in animal feed, as was previously reported [14,39]. Antimicrobial resistance was also detected against amoxicillin and doxycycline suggesting overuse or misuse of these antibiotics [8,40]. Resistance genes, such as *mcr-1*, associated with these phenotypes are often located on plasmids and may be due to co-selection of other antimicrobials rather than directly due to their misuse [41]. Moreover, higher resistance to ciprofloxacin (73.17%) and trimethoprim/sulfamethoxazole (60.98%) deserves attention, because *Salmonella* spp. may cause human infections, and fluoroquinolones are the first-line gut active antimicrobial agents used for the treatment of *Salmonella* infections.

Previous studies have reported that *Salmonella* isolated from poultry in Bangladesh were sensitive to ciprofloxacin [37,42,43]. However, in the present study, *Salmonella* isolates were found to be susceptible to ampicillin, piperacillin/tazobactam, nitrofurantoin, imipenem and amikacin, as was previously reported [39]. These antimicrobial agents are commonly used for therapeutic purposes in veterinary practice and not for feed supplement/growth promotion in Bangladesh, which could explain the susceptibility of the isolates in the current study to such antimicrobial agents. All of the five *mcr-1* positive *Salmonella* isolates in the current study were found to be MDR. Similar findings were also reported on the detection of MDR in *Salmonella* isolates in Bangladesh and different parts of the world [44,45]. The detection of MDR *Salmonella* isolates including colistin resistance is a serious concern in animal and human health due to the high risk of zoonotic transmission of resistant isolates from animals to humans [46].

Since the early 1980s, colistin (also known as polymyxin E) was reported to be widely used in the agricultural and veterinary practice [14], and its overuse has contributed to the initial emergence and spread of *mcr-1* worldwide [14,47]. In this study, an unexpected high rate of phenotypic colistin resistance (92.68% resistant, no isolate was susceptible) was detected in *Salmonella* spp. isolated from chicken samples. While we only screened ten isolates for the underlying genetic determinant (*mcr-1* gene), five isolates were found to be positive for *mcr-1* gene. This alarmingly high rate of colistin resistance in the tested *Salmonella* isolates in chicken suggests that *mcr-1* might be escalating or widespread in food animals in Bangladesh. However, the actual data on antimicrobial use in the tested farms where the samples originated were not available. Therefore, the presence of *mcr-1* gene may suggest imprudent use of colistin and possibly other antimicrobial agents in the poultry industry and livestock production systems in this region, although a recent study reported that 28% of samples obtained from poultry in China harbored *mcr-1* colistin resistance, which is likely to emulate other global antimicrobial resistance [14]. Another study reported an unexpected high prevalence (24.8%) of colistin resistance gene *mcr-1* in retail chicken meat samples in the Netherlands [48]. Further investigations have confirmed the presence of *mcr-1* in *Salmonella* isolates recovered from food animals [49,50]. Various species of *Enterobacteriaceae* carrying *mcr-1* plasmid-mediated resistance have been identified from humans, animals and environments in Asia, Europe, Africa, North America and South America [51]. Furthermore, the proportion of *mcr-1* carriers among colistin-resistant isolates was higher in food-producing animals investigated in Italy [52], Germany [53], UK [54], Poland [55], Bangladesh [56], India [57], Pakistan [58], and South Korea [59]. Interestingly, the detection of the colistin resistance *mcr-1* gene associated with the risk of subsequent transmission to unexposed human populations in southern Vietnam was also reported [60]. Therefore, the high prevalence of the *mcr-1* gene in bacterial isolates of poultry origin in *Enterobacteriaceae* is certainly concerning in a densely populated country such as Bangladesh, where the use of antimicrobial agents in humans and animals may be poorly regulated. Thus, the recent emergence of colistin resistance has triggered an international review and recommendations for restrictions of colistin use in farm animals [47].

As detailed studies on molecular mechanisms of antimicrobial resistance in *Salmonella* isolates of poultry origin in Bangladesh are lacking, we aimed to assess mcr-1 from *Salmonella* samples using an integrative approach ranging from nucleotide sequence analysis, bioinformatics and structural modeling of bacterial genetics. The detection of mcr-1-harbouring *Salmonella* isolates adds new knowledge to the newly emerging issue of colistin resistance and *mcr-1* genes regarding homology, structure and validation of this gene in *Salmonella* isolates. In the present study, multiple sequence alignments for *mcr-1* genes in *Salmonella* spp. isolated from chicken were conducted. In order to avoid hits from very closely related species, retrieved sequences of *Salmonella* species were excluded from the phylogenetic study and those were only aligned with SAUVM MCR-1 proteins. The multiple sequence alignments of SAUVM MCR-1 proteins clearly indicated that they belong to the mobilized colistin resistance (MCR) protein family with putative conserved sites (Supplementary File S2).

In the present study, the detection of colistin resistance gene *mcr-1* in *Salmonella* isolates was confirmed using primer specific PCR (Figure 2) and also by analyzing the possible convergent evolutionary lineages of these newly identified *mcr-1* genes with other previously reported *mcr-1* genes. To address this concern, we conducted phylogenetic analysis which showed a divergent evolutionary pattern between the catalytic domain of LptA of *Nisseria meningitidis* and MCR-1, including MCR-1 like proteins.

The constructed phylogenetic tree provides information about the ancestral origin and diversification of the MCR-1 proteins in different microorganisms which were divided into chromosomally-encoded LptA and plasmid-encoded MCR types, indicating a divergent evolutionary relationship between the LptA and MCR proteins (Figure 3). Further, the MCR protein group was divided into two apparent subgroups; one features MCR-1 proteins, mostly of *E. coli* strains including SAUVM MCR-1, and the other includes a small subclade of MCR-2 with MCR-1 proteins from diverse microorganisms. All of the SAUVM MCR-1 proteins were closely related to the MCR-1 proteins of *E. coli*, sharing the position in the same clade. However, SAUVM MCR-1 and LptA fell into two separate subclades within the tree, which indicated low sequence identity was previously reported [61,62]. The MCR-2 proteins of *E. coli* mostly aligned with MCR-1 proteins of non *E. coli* groups. The Z1140 locus of *E. coli* O157:H7, a member of the phosphatidylethanolamine lipid A transferases lacking a role in colistin resistance, apparently formed an individual clade in the phylogeny, which strengthened the findings.

To increase the structural insight, 3D homology modeling of the five SAUVM MCR-1 proteins was performed using LptA of *Neisseria meningitidis* as a structural template, and five distinct transmembrane helices spanned in the inner membrane region were identified, which was also reported in other studies [47,62]. Molecular interactions between the phosphatidylethanolamine substrate with MCR-1 and LptA were investigated as colistin resistance proteins MCR-1, MCR-2 and LptA were found to share a similar phosphatidylethanolamine lipid substrate-recognizing cavity. Ligand–binding interaction patterns of phosphatidylethanolamine substrate with SAUVM MCR-1 and LptA revealed that both proteins exhibited similar localization of the phosphatidylethanolamine binding sites spanning the 175–195 region in which Phe, Tyr and Ser residues were abundantly found.

## 5. Conclusions

The results of the current study demonstrated the presence of colistin resistance gene *mcr-1* mediated resistance in *Salmonella* spp. isolated from chicken and highlighted the increasing issue of transferable colistin resistance in Bangladesh. The study also showed the in-silico functional analysis and the phylogenetic relationship of the colistin-resistance *mcr-1* genes among *Salmonella* isolates. The results of the present study call for urgent actions to review the extensive use of colistin in poultry production and to limit the imprudent use of colistin and other antimicrobial agents in poultry production systems in Bangladesh.

## Figures and Tables

**Table 1:** Primer sequence for *mcr-1* gene detection in *Salmonella* isolates.

Target Gene	Primer Name	Primer Sequence	Size (bp)
MCR1	MCR1-P1F MCR1-P1R	F: CAGTATGGGATTGCGCAATGATT R: TTATCCATCACGCCTTTTGAGTC	1197
MCR1	MCR1-P2F MCR1-P2R	F: TGTCGATACCGCCAAATACCAAG R: GGAGTGTGCGGTGGGTTTG	799

**Table 2:** Antimicrobial susceptibility profile of *Salmonella* isolates (*n* = 82) in the present study.

Antimicrobial Agents	Susceptible (S)		Intermediate (I)		Resistance (R)		I + R
	Number of Isolates	%	Number of Isolates	%	Number of Isolates	%	
**Penicillins**							
Ampicillin (AMP, 10 μg)	70	85.37	4	4.88	8	9.76	14.63
Amoxycillin/clavulanate (AMC, 20/10 µg)	45	54.88	3	3.66	34	41.46	45.12
Piperacillin/tazobactam (PTZ, 100/10 μg)	69	84.15	6	7.32	8	8.54	15.85
**Aminoglycosides**							
Amikacin (AMK, 30 μg)	53	64.63	7	8.54	22	26.83	35.37
Gentamicin (GEN, 10 μg)	47	57.32	8	9.76	27	32.93	42.68
**Cephalosporins**							
Cefuroxime (CFX, 30 μg)	46	56.10	10	12.20	26	31.71	43.90
Cefuroxime axetil (CFA, 30 μg)	44	53.66	21	25.61	17	20.73	46.34
Ceftriaxone (CTR, 30 μg)	43	52.44	6	7.32	33	40.24	47.56
Cefoperazone/sulbactam (CFS, 75/30 μg)	42	51.22	33	40.24	7	8.54	48.78
Cefepime (CFP, 30 μg)	38	46.34	29	35.37	15	18.29	53.66
**Carbapenems**							
Ertapenem (ETP, 10 μg)	45	54.88	32	39.02	5	6.10	45.12
Imipenem (IMP, 10 μg)	56	68.29	5	6.10	21	25.61	31.71
Meropenem (MPM, 10 μg)	48	58.54	6	7.32	28	34.15	41.46
**Tetracyclines**							
Tigecycline (TIG, 15 μg)	23	28.05	8	9.76	51	62.20	71.95
**Fluoroquinolones**							
Ciprofloxacin (CIP, 5 μg)	16	19.51	6	7.32	60	73.17	80.49
**Nitrofurans**							
Nitrofurantoin (NIT, 300 µg)	60	73.17	8	9.76	14	17.07	26.83
**Polymyxins**							
Colistin (COL, 10 μg)	0	0.00	6	7.32	76	92.68	100.00
**Folate Pathway Inhibitors**							
Trimethoprim/Sulfamethoxazole (SXT, 1.25/23.75 µg)	23	28.05	9	10.98	50	60.98	71.95

% is the percentage (number of isolates/total number of isolates). CLSI zone diameter (in mm) interpretive criteria.

**Table 3:** Multidrug resistance patterns among *mcr-1* positive *Salmonella* spp. isolates in the present study.

	COL	SXT	CIP	TIG	AMC	CTR	GEN *	AMK *	IMP	MPM	NIT
Isolate Number	(10 μg)	(25 μg)	(5 μg)	(15 μg)	(30 μg)	(30 μg)	(10 μg)	(30 μg)	(10 μg)	(10 μg)	(300 μg)
SAUVM S6	R	R	R	R	S	S	S	S	R	R	R
SAUVM S7	R	R	R	S	R	R	R	R	S	S	S
SAUVM S8	R	S	R	R	S	R	R	S	S	S	R
SAUVM S9	R	R	R	S	S	S	S	R	R	R	S
SAUVM S10	R	S	R	R	R	S	R	S	S	S	R

S = susceptible; R = resistance; COL = colistin; SXT = trimethoprim/sulfamethoxazole; CIP = ciprofloxacin; TIG = tigecycline; AMC = amoxicillin/clavulanic acid; CTR = ceftriaxone; GEN = gentamicin; AMK = amikacin; IMP = imipenem; MPM = meropenem; NIT = nitrofurantoin; resistant if, colistin: ≥4; trimethoprim/sulfamethoxazole: ≥80; amoxicillin/clavulanic acid: ≥32; ceftriaxone: ≥4; ciprofloxacin: ≥1; * gentamicin: ≥0.5; * amikacin: ≥0.5; imipenem: ≥4; meropenem: ≥4; nitrofurantoin: ≥128. VITEK 2 systems version: 08.01; control: *E. coli* (ATCC 25922). MIC interpretation guideline/parameter set: copy of global CLSI based + natural resistance. * AES parameter set: copy of global CLSI based + natural resistance [22].

## Data Availability

All data generated and analyzed in the present study were included in the manuscript or available as supplementary files.

## Supplementary Materials

The following are available online at https://www.mdpi.com/2076-2615/11/1/206/s1, Figure S1 (**A**): Structure validation by ERRAT and (**B**): Structure validation by ERRAT.; Figure S2: Structure validation by Verify3D server; Supplementary File S1: Amplicons of 100 bp *invA* gene sequence of *Salmonella* isolates (SAUVM S6 to SAUVM S10), Supplementary File S2: Retrieved sequences of MCR-1 and MCR-1 like proteins, Supplementary File S3: The multiple sequence alignments of SAUVM MCR-1 proteins with putative conserved sites of other *Salmonella* MCR-1., Supplementary File S4: Alignment of MCR-1 proteins.
