# Supplementary material for: Multidrug Antimicrobial Resistance and Molecular Detection of mcr-1 Gene in Salmonella Species Isolated from Chicken
Source: Animals (Basel). 2021 Jan 15;11(1):206. doi: 10.3390/ani11010206 (PMC7829884; doi:10.3390/ani11010206)
Supplement: Supplementary file 1 [file animals-11-00206-s001.zip › Supplementary File S1_14012021.docx]

**Supplementary File S1**

**Nucleotide sequence of *invA* gene of *Salmonella* isolates SAUVM S6, SAUVM S7, SAUVM S8, SAUVM S9, and to SAUVM S10.**

>*Salmonella* isolate_SAUVM S6

TAATGCCAGACGAAAGAGCGTGGTAATTAACAGTACCGCAGGAAACGTTGAAAAACTGAGGATTCTGTCAATGTAGAACGACCCCATAAACACCAATATC

>*Salmonella* isolate_ SAUVM S7

TAATGCCAGACGAAAGAGCGTGGTAATTAACAGTACCGCAGGAAACGTTGAAAAACTGAGGATTCTGTCAATGTAGAACGACCCCATAAACACCAATATC

>*Salmonella* isolate_ SAUVM S8

TAATGCCAGACGAAAGAGCGTGGTAATTAACAGTACCGCAGGAAACGTTGAAAAACTGAGGATTCTGTCAATGTAGAACGACCCCATAAACACCAATATC

>*Salmonella* isolate_ SAUVM S9

TAATGCCAGACGAAAGAGCGTGGTAATTAACAGTACCGCAGGAAACGTTGAAAAACTGAGGATTCTGTCAATGTAGAACGACCCCATAAACACCAATATC

>*Salmonella* isolate_ SAUVM S10

TAATGCCAGACGAAAGAGCGTGGTAATTAACAGTACCGCAGGAAACGTTGAAAAACTGAGGATTCTGTCAATGTAGAACGACCCCATAAACACCAATATC
