# Supplementary material for: Multidrug Antimicrobial Resistance and Molecular Detection of mcr-1 Gene in Salmonella Species Isolated from Chicken
Source: Animals (Basel). 2021 Jan 15;11(1):206. doi: 10.3390/ani11010206 (PMC7829884; doi:10.3390/ani11010206)
Supplement: Supplementary file 1 [file animals-11-00206-s001.zip › Supplementary File S2_14012021.docx]

**Supplementary File S2:**

**Retrieved sequences of MCR-1 and MCR-1 like proteins.**

>*Escherichia coli*_1.17

MMQHTSVWYRRSVSPFVLVASVAVFLTATANLTFFDKISQTYPIADNLGFVLTIAVVLFGAMLLITTLLSSYRYVLKPVL

ILLLIMGAVTSYFTDTYGTVYDTTMLQNALQTDQAETKDLLNAAFIMRIIGLGVLPTLLVAFVKVDYPTWGKGLMRRLGL

IVASLALILLPVVAFSSHYASFFRVHKPLRSYVNPIMPIYSVGKLASIEYKKASAPKDTIYHAKDAVQATKPDMRKPRLV

VFVVGETARADHVSFNGYERDTFPQLAKIDGVTNFSNVTSCGTSTAYSVPCMFSYLGADEYDVDTAKYQENVLDTLDRLG

VSILWRDNNSDSKGVMDKLPKAQFADYKSATNNAICNTNPYNECRDVGMLVGLDDFVAANNGKDMLIMLHQMGNHGPAYF

KRYDEKFAKFTPVCEGNELAKCEHQSLINAYDNALLATDDFIAQSIQWLQTHSNAYDVSMLYVSDHGESLGENGVYLHGM

PNAFAPKEQRSVPAFFWTDKQTGITPMATDTVLTHDAITPTLLKLFDVTADKVKDRTAFIR

>*Escherichia coli*_MCR-1.20

MMQHTSVWYRRSVSPFVLVASVAVFLTATANLTFFDKISQTYPIADNLGFVLTIAVVLFGALLLITTLLSSYRYVLKPVL

ILLLIMGAVTSYFTDTYGTVYDTTMLQNALQTDQAETKDLLNAAFIMRIIGLGVLPSLLVAFVKVDYPTWGKGLMRRLGL

IVASLALILLPVVAFSSHYASFFRVHKPLRSYVNPIMPIYSVGKLASIEYKKASAPKDTIYHAKDAVQATKPDMRKPRLV

VFVVGETARADHVSFNGYERDTFPQLAKIDGVTNFSNVTSCGTSTAYSVPCMFSYLGADEYDVDTAKYQENVLDTLDRLG

VSILWRDNNSDSKGVMDKLPKAQFADYKSATNNAICNTNPYNECRDVGMLVGLDDFVAANNGKDMLIMLHQMGNHGPAYF

KRYDEKFAKFTPVCEGNELAKCEHQSLINAYDNALLATDDFIAQSIQWLQTHSNAYDVSMLYVSDHGESLGENGVYLHGM

PNAFAPKEQRSVPAFFWTDKQTGITPMATDTVLTHDAITPTLLKLFDVTADKVKDRTAFIR

>*Escherichia coli*_MCR-1.7

MMQHTSVWYRRSVSPFVLVASVAVFLTATANLTFFDKISQTYPIADNLGFVLTIAVVLFGAMLLITTLLSSYRYVLKPVL

ILLLIMGAVTSYFTDTYGTVYDTTMLQNALQTDQAETKDLLNAAFIMRIIGLGVLPSLLVAFVKVDYPTWGKGLMRRLGL

IVASLALILLPVVAFSSHYASFFRVHKPLRSYVNPIMPIYSVGKLASIEYKKASTPKDTIYHAKDAVQATKPDMRKPRLV

VFVVGETARADHVSFNGYERDTFPQLAKIDGVTNFSNVTSCGTSTAYSVPCMFSYLGADEYDVDTAKYQENVLDTLDRLG

VSILWRDNNSDSKGVMDKLPKAQFADYKSATNNAICNTNPYNECRDVGMLVGLDDFVAANNGKDMLIMLHQMGNHGPAYF

KRYDEKFAKFTPVCEGNELAKCEHQSLINAYDNALLATDDFIAQSIQWLQTHSNAYDVSMLYVSDHGESLGENGVYLHGM

PNAFAPKEQRSVPAFFWTDKQTGITPMATDTVLTHDAITPTLLKLFDVTADKVKDRTAFIR

>*Escherichia coli*_MCR-1.3

MMQHTSVWYRRSVSPFVLVASVAVFLTATANLTFFDKVSQTYPIADNLGFVLTIAVVLFGAMLLITTLLSSYRYVLKPVL

ILLLIMGAVTSYFTDTYGTVYDTTMLQNALQTDQAETKDLLNAAFIMRIIGLGVLPSLLVAFVKVDYPTWGKGLMRRLGL

IVASLALILLPVVAFSSHYASFFRVHKPLRSYVNPIMPIYSVGKLASIEYKKASAPKDTIYHAKDAVQATKPDMRKPRLV

VFVVGETARADHVSFNGYERDTFPQLAKIDGVTNFSNVTSCGTSTAYSVPCMFSYLGADEYDVDTAKYQENVLDTLDRLG

VSILWRDNNSDSKGVMDKLPKAQFADYKSATNNAICNTNPYNECRDVGMLVGLDDFVAANNGKDMLIMLHQMGNHGPAYF

KRYDEKFAKFTPVCEGNELAKCEHQSLINAYDNALLATDDFIAQSIQWLQTHSNAYDVSMLYVSDHGESLGENGVYLHGM

PNAFAPKEQRSVPAFFWTDKQTGITPMATDTVLTHDAITPTLLKLFDVTADKVKDRTAFIR

>*Escherichia coli*_MCR-1.4

MMQHTSVWYRRSVSPFVLVASVAVFLTATANLTFFDKISQTYPIADNLGFVLTIAVVLFGAMLLITTLLSSYRYVLKPVL

ILLLIMGAVTSYFTDTYGTVYDTTMLQNALQTDQAETKDLLNAAFIMRIIGLGVLPSLLVAFVKVDYPTWGKGLMRRLGL

IVASLALILLPVVAFSSHYASFFRVHKPLRSYVNPIMPIYSVGKLASIEYKKASAPKDTIYHAKDAVQATKPDMRKPRLV

VFVVGETARADHVSFNGYERDTFPQLAKIDGVTNFSNVTSCGTSTAYSVPCMFSYLGADEYDVDTAKYQENVLDTLDRLG

VSILWRDNNSDSKGVMDKLPKAQFADYKSATNNAICNTNPYNECRDVGMLVGLDDFVAANNGKDMLIMLHQMGNHGPAYF

KRYDEKFAKFTPVCEGNELAKCEHQSLINAYDNALLATDNFIAQSIQWLQTHSNAYDVSMLYVSDHGESLGENGVYLHGM

PNAFAPKEQRSVPAFFWTDKQTGITPMATDTVLTHDAITPTLLKLFDVTADKVKDRTAFIR

>*Escherichia coli*_MCR-1.9

MMQHTSVWYRRSVSPFVLVASVAVFLTATANLTFFDKISQTYPIADNLGFVLTIAVVLFGAMLLITTLLSSYRYVLKPVL

ILLLIMGAVTSYFTDTYGTVYDTTMLQNALQTDQAETKDLLNAAFIMRIIGLGVLPSLLVAFVKVDYPTWGKGLMRRLGL

IVASLALILLPVVAFSSHYASFFRVHKPLRSYVNPIMPIYSVGKLASIEYKKASAPKDTIYHAKDAVQATKPDMRKPRLV

VFVVGETARADHVSFNGYERDTFPQLAKIDGVTNFSNVTSCGTSTAYSVPCMFSYLGADEYDVDTAKYQENVLDTLDRLG

VSILWRDNNSDSKGVMDKLPKAQFADYKSATNNAICNTNPYNECRDVGMLVGLDDFVAANNGKDMLIMLHQMGNHGPAYF

KRYDEKFAKFTPACEGNELAKCEHQSLINAYDNALLATDDFIAQSIQWLQTHSNAYDVSMLYVSDHGESLGENGVYLHGM

PNAFAPKEQRSVPAFFWTDKQTGITPMATDTVLTHDAITPTLLKLFDVTADKVKDRTAFIR

>*Escherichia coli*_MCR-1.8

MMRHTSVWYRRSVSPFVLVASVAVFLTATANLTFFDKISQTYPIADNLGFVLTIAVVLFGAMLLITTLLSSYRYVLKPVL

ILLLIMGAVTSYFTDTYGTVYDTTMLQNALQTDQAETKDLLNAAFIMRIIGLGVLPSLLVAFVKVDYPTWGKGLMRRLGL

IVASLALILLPVVAFSSHYASFFRVHKPLRSYVNPIMPIYSVGKLASIEYKKASAPKDTIYHAKDAVQATKPDMRKPRLV

VFVVGETARADHVSFNGYERDTFPQLAKIDGVTNFSNVTSCGTSTAYSVPCMFSYLGADEYDVDTAKYQENVLDTLDRLG

VSILWRDNNSDSKGVMDKLPKAQFADYKSATNNAICNTNPYNECRDVGMLVGLDDFVAANNGKDMLIMLHQMGNHGPAYF

KRYDEKFAKFTPVCEGNELAKCEHQSLINAYDNALLATDDFIAQSIQWLQTHSNAYDVSMLYVSDHGESLGENGVYLHGM

PNAFAPKEQRSVPAFFWTDKQTGITPMATDTVLTHDAITPTLLKLFDVTADKVKDRTAFIR

>*Escherichia coli*_MCR-1.16

MMQHTSVWYRRSVSPFVLVASVAVFLTATANLTFFDKISQTYPIADNLGFVLTIAVVLFGAMLLITTLLSSYRYVLKPVL

ILLLIMGAVTSYFTDTYGTVYDTTMLQNALQTDQAETKDLLNAAFIMRIIGLGVLPSLLVAFVKVDYPTWGKGLMRRLGL

IVASLALILLPVVAFSSHYASFFRVHKPLRSYVNPIMPIYSVGKLASIEYKKASAPKDTIYHAKDAVQATKPDMRKPRLV

VFVVGETARADHVSFNGYERDTFPQLAKIDGVTNFSNVTSCGTSTAYSVPCMFSYLGADEYDVDTAKYQENVLDTLDSLG

VSILWRDNNSDSKGVMDKLPKAQFADYKSATNNAICNTNPYNECRDVGMLVGLDDFVAANNGKDMLIMLHQMGNHGPAYF

KRYDEKFAKFTPVCEGNELAKCEHQSLINAYDNALLATDDFIAQSIQWLQTHSNAYDVSMLYVSDHGESLGENGVYLHGM

PNAFAPKEQRSVPAFFWTDKQTGITPMATDTVLTHDAITPTLLKLFDVTADKVKDRTAFIR

>*Escherichia coli*_MCR-1.5

MMQHTSVWYRRSVSPFVLVASVAVFLTATANLTFFDKISQTYPIADNLGFVLTIAVVLFGAMLLITTLLSSYRYVLKPVL

ILLLIMGAVTSYFTDTYGTVYDTTMLQNALQTDQAETKDLLNAAFIMRIIGLGVLPSLLVAFVKVDYPTWGKGLMRRLGL

IVASLALILLPVVAFSSHYASFFRVHKPLRSYVNPIMPIYSVGKLASIEYKKASAPKDTIYHAKDAVQATKPDMRKPRLV

VFVVGETARADHVSFNGYERDTFPQLAKIDGVTNFSNVTSCGTSTAYSVPCMFSYLGADEYDVDTAKYQENVLDTLDRLG

VSILWRDNNSDSKGVMDKLPKAQFADYKSATNNAICNTNPYNECRDVGMLVGLDDFVAANNGKDMLIMLHQMGNHGPAYF

KRYDEKFAKFTPVCEGNELAKCEHQSLINAYDNALLATDDFIAQSIQWLQTYSNAYDVSMLYVSDHGESLGENGVYLHGM

PNAFAPKEQRSVPAFFWTDKQTGITPMATDTVLTHDAITPTLLKLFDVTADKVKDRTAFIR

>*Escherichia coli*_MCR-1.22

MMQHTSVWYRRSVSPFVLVASVAVFLTATANLTFFDKISQTYPIADNLGFVLTIAVVLFGAMLLITTLLSSYRYVLKPVL

ILLLIMGAVTSYFTDTYGTVYDTTMLQNALQTDQAETKDLLNAAFIMRIIGLGVLPSLLVAFVKVDYPTWGKGLMRRLGL

IVASLALILLPVVAFSSHYASFFRVHKPLRSYVNPIMPIYSVGKLASIEYKKASAPKDTIYHAKDAVQATKPDMRKPRLV

VFVVGETARADHVSFNGYERDTFPQLAKIDGVTNFSNVTSCGTSTAYSVPCMFSYLGADEYDVDTAKYQENVLDTLDRLG

VSILWRDNNSDSKGVMDKLPKAQFADYKSATNNAICNTNPYNECRDVGMLVGLDDFVAANNGKDMLIMLHQMGNHGPAYF

KRYDEKFAKFTPVCEGNELAKCEHQFLINAYDNALLATDDFIAQSIQWLQTHSNAYDVSMLYVSDHGESLGENGVYLHGM

PNAFAPKEQRSVPAFFWTDKQTGITPMATDTVLTHDAITPTLLKLFDVTADKVKDRTAFIR

>*Escherichia coli*_MCR-1.12

MMHHTSVWYRRSVSPFVLVASVAVFLTATANLTFFDKISQTYPIADNLGFVLTIAVVLFGAMLLITTLLSSYRYVLKPVL

ILLLIMGAVTSYFTDTYGTVYDTTMLQNALQTDQAETKDLLNAAFIMRIIGLGVLPSLLVAFVKVDYPTWGKGLMRRLGL

IVASLALILLPVVAFSSHYASFFRVHKPLRSYVNPIMPIYSVGKLASIEYKKASAPKDTIYHAKDAVQATKPDMRKPRLV

VFVVGETARADHVSFNGYERDTFPQLAKIDGVTNFSNVTSCGTSTAYSVPCMFSYLGADEYDVDTAKYQENVLDTLDRLG

VSILWRDNNSDSKGVMDKLPKAQFADYKSATNNAICNTNPYNECRDVGMLVGLDDFVAANNGKDMLIMLHQMGNHGPAYF

KRYDEKFAKFTPVCEGNELAKCEHQSLINAYDNALLATDDFIAQSIQWLQTHSNAYDVSMLYVSDHGESLGENGVYLHGM

PNAFAPKEQRSVPAFFWTDKQTGITPMATDTVLTHDAITPTLLKLFDVTADKVKDRTAFIR

>*Escherichia coli*_MCR-1.21

MMQHTSVWYRRSVSPFVLVASVAVFLTATANLTFFDKISQTYPIADNLGFVLTIAVVLFGAMLLITTLLSSYRYVLKPVL

ILLLIMGAVTSYFTDTYGTVYDTTMLQNALQTDQAETKDLLNAAFIMRIIGLGVLPSLLVAFVKVDYPTWGKGLMRRLGL

IVASLALILLPVVAFSSHYASFFRVHKPLRSYVNPIMPIYSVGKLASIEYKKASAPKDTIYHAKDAVQATKPDMRKPRLV

VFVVGETARADHVSFNGYERDTFPQLAKIDGVTNFSNVTSCGTSTAYSVPCMFSYLGADEYDVDTAKYQENVLDTLDRLG

VSILWRDNNSDSKGVMDKLPKAQFADYKSATNNAICNTNPYNECRDVGMLVGLDDFVAANNGKDMLIMLHQMGNHGPAYF

KRYDEKFAKFTSVCEGNELAKCEHQSLINAYDNALLATDDFIAQSIQWLQTHSNAYDVSMLYVSDHGESLGENGVYLHGM

PNAFAPKEQRSVPAFFWTDKQTGITPMATDTVLTHDAITPTLLKLFDVTADKVKDRTAFIR

>*Escherichia coli*_MCR-1.11

MMQHTSVVWYRRSVSPFVLVASVAVFLTATANLTFFDKISQTYPIADNLGFVLTIAVVLFGAMLLITTLLSSYRYVLKPV

LILLLIMGAVTSYFTDTYGTVYDTTMLQNALQTDQAETKDLLNAAFIMRIIGLGVLPSLLVAFVKVDYPTWGKGLMRRLG

LIVASLALILLPVVAFSSHYASFFRVHKPLRSYVNPIMPIYSVGKLASIEYKKASAPKDTIYHAKDAVQATKPDMRKPRL

VVFVVGETARADHVSFNGYERDTFPQLAKIDGVTNFSNVTSCGTSTAYSVPCMFSYLGADEYDVDTAKYQENVLDTLDRL

GVSILWRDNNSDSKGVMDKLPKAQFADYKSATNNAICNTNPYNECRDVGMLVGLDDFVAANNGKDMLIMLHQMGNHGPAY

FKRYDEKFAKFTPVCEGNELAKCEHQSLINAYDNALLATDDFIAQSIQWLQTHSNAYDVSMLYVSDHGESLGENGVYLHG

MPNAFAPKEQRSVPAFFWTDKQTGITPMATDTVLTHDAITPTLLKLFDVTADKVKDRTAFIR

>*Escherichia coli*_MCR-2.1

MTSHHSWYRYSINPFVLMGLVALFLAATANLTFFEKAMAVYPVSDNLGFIISMAVAVMGAMLLIVVLLSYRYVLKPVLIL

LLIMGAVTSYFTDTYGTVYDTTMLQNAMQTDQAESKDLMNLAFFVRIIGLGVLPSVLVAVAKVNYPTWGKGLIQRAMTWG

VSLVLLLVPIGLFSSQYASFFRVHKPVRFYINPITPIYSVGKLASIEYKKATAPTDTIYHAKDAVQTTKPSERKPRLVVF

VVGETARADHVQFNGYGRETFPQLAKVDGLANFSQVTSCGTSTAYSVPCMFSYLGQDDYDVDTAKYQENVLDTLDRLGVG

ILWRDNNSDSKGVMDKLPATQYFDYKSATNNTICNTNPYNECRDVGMLVGLDDYVSANNGKDMLIMLHQMGNHGPAYFKR

YDEQFAKFTPVCEGNELAKCEHQSLINAYDNALLATDDFIAKSIDWLKTHEANYDVAMLYVSDHGESLGENGVYLHGMPN

AFAPKEQRAVPAFFWSNNTTFKPTASDTVLTHDAITPTLLKLFDVTAGKVKDRAAFIQ

>*Escherichia coli*_MCR-2.3

MTSHHSWYRYSINPFVLMGLVALFLAATANLTFFEKAMAVYPVSDNLGFIISMAVAVMGAMLLIVVLLSYRYVLKPVLIL

LLIMGAVTSYFTDTYGTVYDTTMLQNAMQTDQAESKDLMNLAFFVRIIGLGVLPSLLVAVAKVGYPTWGKSLIQRAMTWG

VSLVLLLVPIGLFSSQYASFFRVHKPVRFYINPITPIYSVGKLASIEYKKATAPTDTIYHAKDAVQTTKPSERKPRLVVF

VVGETARADHVQFNGYSRETFPQLAKVDGLANFSQVTSCGTSTAYSVPCMFSYLGQDDYDVDTAKYQENVLDTLDRLGVG

ILWRDNNSDSKGVMDKLPATQYFDYKSATNNTICNTNPYNECRDVGMLVGLDDYVSANNGKDMLIMLHQMGNHGPAYFKR

YDEQFTKFTPVCEGNELAKCEHQSLINAYDNALLATDDFIAKSIDWLKTHEANYDVAMLYVSDHGESLGENGVYLHGMPN

AFAPKEQRAVPVFFWSNNTTFKPTASDTALTHDAITPTLLKLFDVTADKVKDRTAFIQ

>*Gammaproteobacteria*_MCR-1.1

MMQHTSVWYRRSVSPFVLVASVAVFLTATANLTFFDKISQTYPIADNLGFVLTIAVVLFGAMLLITTLLSSYRYVLKPVL

ILLLIMGAVTSYFTDTYGTVYDTTMLQNALQTDQAETKDLLNAAFIMRIIGLGVLPSLLVAFVKVDYPTWGKGLMRRLGL

IVASLALILLPVVAFSSHYASFFRVHKPLRSYVNPIMPIYSVGKLASIEYKKASAPKDTIYHAKDAVQATKPDMRKPRLV

VFVVGETARADHVSFNGYERDTFPQLAKIDGVTNFSNVTSCGTSTAYSVPCMFSYLGADEYDVDTAKYQENVLDTLDRLG

VSILWRDNNSDSKGVMDKLPKAQFADYKSATNNAICNTNPYNECRDVGMLVGLDDFVAANNGKDMLIMLHQMGNHGPAYF

KRYDEKFAKFTPVCEGNELAKCEHQSLINAYDNALLATDDFIAQSIQWLQTHSNAYDVSMLYVSDHGESLGENGVYLHGM

PNAFAPKEQRSVPAFFWTDKQTGITPMATDTVLTHDAITPTLLKLFDVTADKVKDRTAFIR

>*Raoultella planticola*_MCR-1.3

MMQHTSVWYRRSVSPLVLVASVAVFLTATANLTFFDKISQTYPIADNLGFVLTIAVVLFGAMLLITTLLSSYRYVLKPVL

ILLLIMGAVTSYFTDTYGTVYDTTMLQNALQTDQAETKDLLNAAFIMRIIGLGVLPSLLVAFVKVDYPTWGKGLMRRLGL

IVASLALILLPVVAFSSHYASFFRVHKPLRSYVNPIMPIYSVGKLASIEYKKASAPKDTIYHAKDAVQATKPDMRKPRLV

VFVVGETARADHVSFNGYERDTFPQLAKIDGVTNFSNVTSCGTSTAYSVPCMFSYLGADEYDVDTAKYQENVLDTLDRLG

VSILWRDNNSDSKGVMDKLPKAQFADYKSATNNAICNTNPYNECRDVGMLVGLDDFVAANNGKDMLIMLHQMGNHGPAYF

KRYDEKFAKFTPVCEGNELAKCEHQSLINAYDNALLATDDFIAQSIQWLQTHSNAYDVSMLYVSDHGESLGENGVYLHGM

PNAFAPKEQRSVPAFFWTDKQTGITPMATDTVLTHDAITPTLLKLFDVTADKVKDRTAFIR

>*Citrobacter amalonaticus*_MCR-1

MMQHTSVWYRRSVSPFVLVASVAVFLTATANLTFFDKISQTYPIADNLGFVLTIAVVLFGAMLLITTLLSSYRYVLKPVL

ILLLIMGAVTSYFTDTYGTVYDTTMLQNALQTDQAETKDLLNAAFIMRIIGLGVLPSLLVAFVKVDYPTWGKGLMRRLGL

IVASLALILLPVVAFSSHYASFFRVHKPLRSYVNPIMPIYSVGKLASIEYKKASAPKDTIYHAKDAVQATKPDMRKPRLV

VFVVGETARADHVSFNGYERDTFPQLAKIDGVTNFSNVTSCGTSTAYSVPCMFSYLGADEYDVDTAKYQENVLDTLDRLG

VSILWRDNNSDSKGVMDKLPKAQFADYKSATNNAICNTNPYNECRDVGMLVGLDDFVAANNGKDMLIMLHQMGNHGPAYF

KRYDEKFAKFTPVCEGNELAKCEHQSLINAYDNALLATDDFIAQSIQWLQTYSNAYDVSMLYVSDHGESLGENGVYLHGM

PNAFAPKEQRSVPAFFWTDKQTGITPMATDTVLTHDAITPTLLKLFDVTADKVKDRTAFIR

>*Klebsiella pneumoniae_*MCR-1.14

MMQHTSVWYRRSVSPFVLVASVAVFLTATANLTFFDKVSQTYPIADNLGFVLTIAVVLFGAMLLITTLLSSYRYVLKPVL

ILLLIMGAVTSYFTDTYGTVYDTTMLQNALQTDQAETKDLLNAAFIMRIIGLGVLPSLLVAFVKVDYPTWGKGLMRRLGL

IVASLALILLPVVAFSSHYASFFRVHKPLRSYVNPIIPIYSVGKLASIEYKKASAPKDTIYHAKDAVQATKPDMRKPRLV

VFVVGETARADHVSFNGYERDTFPQLAKIDGVTNFSNVTSCGTSTAYSVPCMFSYLGADEYDVDTAKYQENVLDTLDRLG

VSILWRDNNSDSKGVMDKLPKAQFADYKSATNNAICNTNPYNECRDVGMLVGLDDFVAANNGKDMLIMLHQMGNHGPAYF

KRYDEKFAKFTPVCEGNELAKCEHQSLINAYDNALLATDDFIAQSIQWLQTHSNAYDVSMLYVSDHGESLGENGVYLHGM

PNAFAPKEQRSVPAFFWTDKQTGITPMATDTVLTHDAITPTLLKLFDVTADKVKDRTAFIR

>*Enterobacteriaceae*_MCR-1.2

MMLHTSVWYRRSVSPFVLVASVAVFLTATANLTFFDKISQTYPIADNLGFVLTIAVVLFGAMLLITTLLSSYRYVLKPVL

ILLLIMGAVTSYFTDTYGTVYDTTMLQNALQTDQAETKDLLNAAFIMRIIGLGVLPSLLVAFVKVDYPTWGKGLMRRLGL

IVASLALILLPVVAFSSHYASFFRVHKPLRSYVNPIMPIYSVGKLASIEYKKASAPKDTIYHAKDAVQATKPDMRKPRLV

VFVVGETARADHVSFNGYERDTFPQLAKIDGVTNFSNVTSCGTSTAYSVPCMFSYLGADEYDVDTAKYQENVLDTLDRLG

VSILWRDNNSDSKGVMDKLPKAQFADYKSATNNAICNTNPYNECRDVGMLVGLDDFVAANNGKDMLIMLHQMGNHGPAYF

KRYDEKFAKFTPVCEGNELAKCEHQSLINAYDNALLATDDFIAQSIQWLQTHSNAYDVSMLYVSDHGESLGENGVYLHGM

PNAFAPKEQRSVPAFFWTDKQTGITPMATDTVLTHDAITPTLLKLFDVTADKVKDRTAFIR

>*Klebsiella pneumoniae*_MCR-1.15

MQHTSVWYRRSVSPFVLVASVAVFLTATANLTFFDKISQTYPIADNLGFVLTIAVVLFGAMLLITTLLSSYRYVLKPVLI

LLLIMGAVTSYFTDTYGTVYDTTMLQNALQTDQAETKDLLNAAFIMRIIGLGVLPSLLVAFVKVDYPTWGKGLMRRLGLI

VASLALILLPVVAFSSHYASFFRVHKPLRSYVNPIMPIYSVGKLASIEYKKASAPKDTIYHAKDAVQATKPDMRKPRLVV

FVVGETARADHVSFNGYERDTFPQLAKIDGVTNFSNVKSCGTSTAYSVPCMFSYLGADEYDVDTAKYQENVLDTLDRLGV

SILWRDNNSDSKGVMDKLPKAQFADYKSATNNAICNTNPYNECRDVGMLVGLDDFVAANNGKDMLIMLHQMGNHGPAYFK

RYDEKFAKFTPVCEGNELAKCEHQSLINAYDNALLATDDFIAQSIQWLQTHSNAYDVSMLYVSDHGESLGENGVYLHGMP

NAFAPKEQRSVPAFFWTDKQTGITPMATDTVLTHDAITPTLLKLFDVTADKVKDRTAFIR

>*Moraxella* sp. MSG13-C03_MCR-1.10

MVQHTSVWYRCSVSPFVLVASVSVFLTATANLTFFDKISQTYPIADNLGFVLTIAVVLFGAMLLITTLLSSYRYVLKPVL

ILLLIMGAVTSYFTDTYGTVYDTTMLQNALQTDQAETKDLLNAAFIMRIIGLGVLPSLLVAFVKVDYPTWGKGLVRRLGL

IVASLALILLPVVAFSSHYASFFRVHKPLRSYVNPIMPIYSVGKLASIEYKKASAPKDTIYHAKDAVQATKPDTRKPRLV

VFVVGETARADHVSFNGYERDTFPQLAKIDGVTNFSNVTSCGTSTAYSVPCMFSYLGADEYDVDTAKYQENVLDTLDRLG

VSILWRDNNSDSKGVMDKLPKAQFADYKSATNNTICNTNPYNECRDVGMLVGLDDFVAANNGKDMLIMLHQMGNHGPAYF

KRYDEKFAKFTPVCEGNELAKCEHQSLINAYDNALLATDDFITQSIQWLQTHSNAYDVSMLYVSDHGESLGENGVYLHGM

PNAFAPKEQRSVPAFFWTDKQTGITPMATDTVLTHDAITPTLLKLFDVTADKVKDRTAFIR

>*Citrobacter braakii*_MCR-1

MWYRRSVSPFVLVASVAVFLTATANLTFFDKISQTYPIADNLGFVLTIAVVLFGAMLLITTLLSSYRYVLKPVLILLLIM

GAVTSYFTDTYGTVYDTTMLQNALQTDQAETKDLLNAAFIMRIIGLGVLPSLLVAFVKVDYPTWGKGLMRRLGLIVASLA

LILLPVVAFSSHYASFFRVHKPLRSYVNPIMPIYSVGKLASIEYKKASAPKDTIYHAKDAVQATKPDMRKPRLVVFVVGE

TARADHVSFNGYERDTFPQLAKIDGVTNFSNVTSCGTSTAYSVPCMFSYLGADEYDVDTAKYQENVLDTLDRLGVSILWR

DNNSDSKGVMDKLPKAQFADYKSATNNAICNTNPYNECRDVGMLVGLDDFVAANNGKDMLIMLHQMGNHGPAYFKRYDEK

FAKFTPVCEGNELAKCEHQSLINAYDNALLATDDFIAQSIQWLQTHSNAYDVSMLYVSDHGESLGENGVYLHGMPNAFAP

KEQRSVPAFFWTDKQTGITPMATDTVLTHDAITPTLLKLFDVTADKVKDRTAFIR

>*Escherichia albertii*_MCR-1

VSPFVLVASVAVFLTATANLTFFDKISQTYPIADNLGFVLTIAVVLFGAMLLITTLLSSYRYVLKPVLILLLIMGAVTSY

FTDTYGTVYDTTMLQNALQTDQAETKDLLNAAFIMRIIGLGVLPSLLVAFVKVDYPTWGKGLMRRLGLIVASLALILLPV

VAFSSHYASFFRVHKPLRSYVNPIMPIYSVGKLASIEYKKASAPKDTIYHAKDAVQATKPDMRKPRLVVFVVGETARADH

VSFNGYERDTFPQLAKIDGVTNFSNVTSCGTSTAYSVPCMFSYLGADEYDVDTAKYQENVLDTLDRLGVSILWRDNNSDS

KGVMDKLPKAQFADYKSATNNAICNTNPYNECRDVGMLVGLDDFVAANNGKDMLIMLHQMGNHGPAYFKRYDEKFAKFTP

VCEGNELAKCEHQSLINAYDNALLATDDFIAQSIQWLQTHSNAYDVSMLYVSDHGESLGENGVYLHGMPNAFAPKEQRSV

PAFFWTDKQTGITPMATDTVLTHDAITPTLLKLL

>*Providencia stuartii*_MCR-1

ATANLTFFDKISQTYPIADNLGFVLTIAVVLFGAMLLITTLLSSYRYVLKPVLILLLIMGAVTSYFTDTYGTVYDTTMLQ

NALQTDQAETKDLLNAAFIMRIIGLGVLPSLLVAFVKVDYPTWGKGLMRRLGLIVASLALILLPVVAFSSHYASFFRVHK

PLRSYVNPIMPIYSVGKLASIEYKKASAPKDTIYHAKDAVQATKPDMRKPRLVVFVVGETARADHVSFNGYERDTFPQLA

KIDGVTNFSNVTSCGTSTAYSVPCMFSYLGADEYDVDTAKYQENVLDTLDRLGVSILWRDNNSDSKGVMDKLPKAQFADY

KSATNNAICNTNPYNECRDVGMLVGLDDFVAANNGKDMLIMLHQMGNHGPAYFKRYDEKFAKFTPVCEGNELAKCEHQSL

INAYDNALLATDDFIAQSIQWLQTHSNAYDVSMLYVSDHGESLGENGVYLHGMPNAFAPKEQRSVPAFFWTDKQTGITPM

ATDTVLTHDAITPTLLKL

>*Moraxella* sp. MSG47-C17_MCR1

MTQHSPWYRRPVNPYLLMSVVALFLSATANLTFFDKITNTYPMAQNAGFVISTALVLFGAMLLITVLLSYRYVLKPVLIL

LLIMGAVTSYFTDTYGTVYDTTMLQNALQTDQAESKDLMNMAFFVRIIGLGVLPSILVAWVKVDYPTLGKSLIQRAMTWG

VAVVMALVPILAFSSHYASFFREHKPLRSYVNPVMPIYSVGKLASIEYKKATAPKDTIYHAKDAVQTTTPAERKPRLVVF

VVGETARADHVQFNGYSRETFPQLAKIDNLANFSQVTSCGTSTAYSVPCMFSYLGQDDYDVDTAKYQENVLDTLDRLGVG

ILWRDNNSDSKGVMDKLPASQYFDYKSATNNTICNTNPYNECRDVGMLVGLDDYVSTNQGKDMLIMLHQMGNHGPAYFKR

YDEQFAKYTPVCEGNELAKCEHQSLINAYDNALLATDDFIAKSIDWLKTHQANYDVAMLYVSDHGESLGENGVYLHGMPN

AFAPKEQRAVPAFFWSNNPSFTPTASDTVLTHDAITPTLLKLFDVTADKVKDRTAFIR

>*Moraxella pluranimalium*_MCR-2.2

MTSQHSWYRYSINPFVLMGLVALFLAATANLTFFEKAMAVYPVSDNLGFIISMAVALMGAMLLIVVLLSYRYVLKPVLIL

LLIMGAVTSYFTDTYGTVYDTTMLQNAMQTDQAESKDLMNLAFFVRIIGLGVLPSVLVAFAKVNYPTWGKGLIQRAMTWG

VSLVLLLVPIGLFSSQYASFFRVHKPVRFYINPITPIYSVGKLASIEYKKATAPTDTIYHAKDAVQTTKPSERKPRLVVF

VVGETARADHVQFNGYGRETFPQLAKVDGLANFSQVTSCGTSTAYSVPCMFSYLGQDDYDVDTAKYQENVLDTLDRLGVD

ILWRDNNSDSKGVMDKLPTTQYFDYKSATNNTICNTNPFNECRDVGMLVGLDDYVSANNGKDMLIMLHQMGNHGPAYFKR

YDEQFAKFTPVCEGNELAKCEHQSLINAYDNALLATDDFIAKSIDWLKTHEANYDVAMLYVSDHGESLGENGVYLHGMPN

AFAPKEQRAVPAFFWSNNTTFKPTASDTVLTHDAITPTLLKLFDVTADKVKDRTAFIQ

>*Moraxella osloensis_*MCR-1

MVHLDKVSNRMSVNNSRWLAWRQQGINAYVMMGIVALFLTLTANITFFDKATEVYPFAQHIGFIGSLPLVLFGVMLLVIV

LLSYRYTLKAVLIFLLLTAAVTAYFTDTYGTVYDVNMLQNALQTDKAESADLFNVNFILRILLLGVLPSVWVAWQKVTFP

PIKRSILQRGLTYLVSLGLVVLPILAMSKNYASFFREHKQLRSYTNPATPVYALGKLASIQLKQAQAPKTQIMHATDAVQ

VSNPTTRKPKLVVFVVGETARGDHVQLNGYNRTTFPQMAATAGVTNFNQVIACGTSTAYSVPCMFSYVGMKDYDVDTANY

QENVLDTLHRLKVNILWRDNNSSSKGVTNRLPAADFVDYKTARNNTMCNTNPYGECRDVGMLVGLDDYVKQQANQNTLNQ

DTLIVLHQMGNHGPAYFKRYDKQFEKFTPVCQSNELAKCDPQSVINAFDNALLATDDFLAKTVNWLDKYDSTHQVAMLYV

SDHGESLGENGIYLHGMPYKIAPKAQKHVASMFWAGKHSGIQAVPSNTELTHDAITPTLLKLFDVRAQTVQGKPLFIK

>*Moraxellaceae bacterium*_MCR-1

MSVNNSRWVAWRQQGINAYVMMGIVALFLTLTANVTFFDKATEVYPFAQHIGFIGSLPLVLCGVMLLVIVLLSYRYTLKA

VLIFLLLTAAVTAYFTDTYGTVYDVNMLQNALQTDKAESADLFNVNFILRILLLGVLPSVWVVWQKVTFPPIKRSILQRG

LTYLVSLGLVVLPILAMSKNYASFFREHKQLRSYTNPATPVYALGKLASIQLKQAQAPKTQIMHATDAVQVSNPSTRKPK

LVVLVVGETARGDHVQLNGYNRTTFPQMAATAGVTNFNQVIACGTSTAYSVPCMFSYVGMKDYDVDTANYQENVLDTLHR

LKVNILWRDNNSSSKGVTNRLPAADFVDYKTARNNTMCNTNPYGECRDVGMLVGLDDYVKQLANQNTLNQDTLIVLHQMG

NHGPAYFKRYDKQFEKFTPVCQSNELAKCDPQSVINAFDNALLATDDFLAKTVNWLGKYDSTHQVAMLYVSDHGESLGEN

GIYLHGMPYKIAPKAQKHVASMFWAGKHSGIQAVPSNTELTHDAITPTLLKLFDVRAQTVQGKPLFIK

>*Enhydrobacter aerosaccus*_MCR-1

MSVNNSRWVAWRQQGINTKSVSTQGISTQGINAYVMMGIVALFLTLTANITFFDKATAVYPFAQHIGFIGSLPLVLCGVM

LLVIVLLSYRYTLKVVLIFLLLTAAVTAYFTDTYGTVYDVNMLQNALQTDKAESADLFNVNFILRILLLGVLPSVWVAWQ

KVTFPPIKRSILQRGLTYLVSIGLVVLPILAMSKNYASFFREHKQLRSYTNPATPVYALGKLASIQLKQAQAPKTQIMHA

TDAVQVSNPTTRKPKLVVLVVGETARGDHVQLNGYNRTTFPQMAATAGVTNFNQVIACGTSTAYSVPCMFSYVGMKDYDV

DTANYQENVLDTLNRLKVNILWRDNNSSSKGVTNRLPAADFVDYKTARNNTMCNTNPYGECRDVGMLVGLDDYVKQLANQ

NTLNQDTLIVLHQMGNHGPAYFKRYDKQFEKFTPVCQSNELAKCDPQSVINAFDNALLATDDFLAKTVNWLDKYDSTHQV

AMLYVSDHGESLGENGIYLHGMPYKIAPKAQKHVASMFWAGKHSGIQAVPSNTELTHDAITPTLLKLFDVRAQTVQGKPL

FIK

>*Leucothrix* sp. C3212_MCR-1

MMGIVALFLTLTANITFFDKATEVYPFAQHIGFIGSLPLVLFGVMLLVIVLLSYRYTLKAVLIFLLLTAAVTAYFTDTYG

TVYDVNMLQNALQTDKAESADLFNVNFILRILLLGVLPSVWVAWQKVTFPPIKRSLLQRGLTYLVSLGLVVLPILAMSKN

YASFFREHKQLRSYTNPATPVYALGKLASIQLKQAQAPKTQIMHATDAVQVKNPTTRKPKLMVLVVGETARGDHVQLNGY

NRTTFPQMAAMAGVTNFNQVIACGTSTAYSVPCMFSYVGMKDYDVDTANYQENVLDTLHRLKVNILWRDNNSSSKGVTNR

LPAENFVDYKTARNNTMCNTNPYGECRDVGMLVGLDDYVKQQANQDTLNQDTLIVLHQMGNHGPAYFKRYDKQFEKFTPV

CQTNELAKCDPQSVINAFDNALLATDDFLAKTVNWLDKYDSTHQVAMLYVSDHGESLGENGIYLHGMPYKIAPKAQKHVA

SMFWAGKHSGIQAVPSNTELTHDAITPTLLKLFDVRAQTVQGKPLFIK

>*Bacterium* M00.F.Ca.ET.230.01.1.1_MCR-1

MMGIVALFLTLTANVTFFDKATEVYPFAQHIGFIGSLPLVLCGVMLLVIVLLSYRYTLKAVLIFLLLTAAVTAYFTDTYG

TVYDVNMLQNALQTDKAESADLFNVNFILRILLLGVLPSVWVAWQKVTFPPIKRSLLQRGLTYLVSLGLVVLPILAMSKN

YASFFREHKQLRSYTNPATPVYALGKLASIQLKQAQAPKTQIMHATDAVQVSNPTTRKPKLVVLVVGETARGDHVQLNGY

NRTTFPQMAATAGVTNFNQVIACGTSTAYSVPCMFSYVGMKDYDVDTANYQENVLDTLHRLKVNILWRDNNSSSKGVTNR

LPAADFVDYKTARNNTMCNTNPYGECRDVGMLVGLDDYVKQQANQNTLNQDTLIVLHQMGNHGPAYFKRYDKQFEKFTPV

CQSNELAKCDPQSVINAFDNALLATDDFLAKTVNWLGKYDSTHQVAMLYVSDHGESLGENGIYLHGMPYKIAPKAQKHVA

SMFWAGKHSGIQAVPSNTELTHDAITPTLLKLFDVRAQTVQGKPLFIK

>*Enhydrobacter* sp. H5_MCR-1

MMGIVALFLTLSANVTFFDKATAVYPFAQHIGFIGSLPLVLCGVMLLVIVLLSYRYTLKVVLIFLLLTAAVTAYFTDTYG

TVYDVNMLQNALQTDKAESADLFNVNFILRILLLGVLPSVWVAWQKVTFPPIKRSILQRGLTYLVSLGLVVLPILAMSKN

YASFFREHKQLRSYTNPATPVYALGKLASIQLKQAQAPKTQIMHATDAVQVSNPTTRKPKLVVLVVGETARADHVQLNGY

NRTTFPQMAATAGVTNFNQVIACGTSTAYSVPCMFSYVGMKDYDVDTANYQENVLDTLNRLKVNILWRDNNSSSKGVTNR

LPAENFVDYKTARNNTMCNTNPYGECRDVGMLVGLDDYVKQQANQNTLNQDTLIVLHQMGNHGPAYFKRYDKQFEKFTPV

CQSNELAKCDPQSVINAFDNALLATDDFLAKTVNWLDKYDSTHQVAMLYVSDHGESLGENGIYLHGMPYKIAPKAQKHVA

SMFWAGKHSGIQAVPSNTELTHDAITPTLLKLFDVRAQTVQGKPLFIQ

>*Moraxella porci_*MCR-1

MLNFLHPKHRSINPYLLMLIVAVFLTISANVTFFKQVVLVYPIADHLLFVASLTVVLCGVLALVIGLFSYRYTLKFVLIF

MIMVAAITSYFTDTYGTVYDTNMLQNALQTDSSETKDLLNLGFLVRILLLGVLPSLLILKLPVHFANFKTNAFQRLGYLL

LSLGLILVPILSFSEAFASFFREHKPLRSYTNPAMPIYAVGKLASIEYKKATAPKDLTYHAKDAAQTTTSTQRKPKLIVM

VVGETARADHVSLNGYSKDTFVQLSSMDHLTSFKNVISCGTSTAYSVPCMFSYLGADDYNVDTANYHENSLDTLHRLGVN

VLWRDNNSDSKGVMDKLPADLYQNYKTSDLNHECTNAHQECRDIGMLIGLDDYVAQTAKNSNQDVLIVLHQMGNHGPAYY

KRYNEAFEKFTPVCRDNDLAKCDTSHVINAYDNALVATDDFLKQTIDWLKTHQASHDVTLLYVSDHGESLGENGVYLHGM

PNTFAPKAQKHVAAFLWTANPAIHAVSNQAPLTHDAITPTLLRLFDVKTKATENQAMFIE

>*Neisseria*_LptA

MIKPNLRPKLGSSALIAFLSLYSSLVLNYAFFAKVVELRPFNDTGADIFLYTMPVVLFFLSNFVFHVIALPFVHKVLIPL

ILVISAAVSYQEIFFNIYFNKSMLNNVLQTTAAESARLITPGYVLWIVCLGVLPALAYIAVKVKYRVWYKELLTRLVLAA

VSFLCALGIAMLQYQDYASFFRNNKSVTHLIVPSNFIGAGVSKYKDWKRSNIPYTQLDMAVVQNRPAGSLRRFVVLVVGE

TTRAANWGLNGYSRQTTPLLAARGDEIVNFPQVRSCGTSTAHSLPCMFSTFDRTDYDEIKAEHQDNLLDIVQRAGVEVTW

LENDSGCKGVCGKVPNTDVTSLNLPEYCRNGECLDNILLTKFDEALNKNDKDAVLILHTIGSHGPTYYERYTEAERKFTP

TCDTNEIDKCARATLVNTYDNTVLYVDQFIDKVIRKLENRDDLESAVYYVSDHGESLGENGMYLHAAPYAIAPSGQTHIP

MVMWFSKAFRQHGGIDFQCLKQKAAENEYSHDHYFSTVLGLMDISNSQTYRKEMDILAACRRPR

>*Neisseria gonorrhoeae*_LptA

MIKPNLRPKLGSSALIAFLSLYSSLVLNYAFFAKVVELRPFNDTGADIFLYTMPVVLFFLSNFVFHVIALPFVHKVLIPL

ILVISAAVSYQEIFFNIYFNKSMLNNVLQTTAAESARLITPGYVLWIVCLGVLPALAYIAVKVKYRVWYKELLTRLVLAA

VSFLCALGIAMLQYQDYASFFRNNKSVTHLIVPSNFIGAGVSKYKDWKRSNIPYTQLDMAVVQTRPAGSLRRFVVLVVGE

TTRAANWGLNGYSRQTTPLLAARGDEIVNFPQVRSCGTSTAHSLPCMFSTFDRTDYDEIKAEHQDNLLDIVQRAGVEVTW

LENDSGCKGVCGKVPNTDVTSLNLPEYCRNGECLDNILLTKFDEALNKNDKDAVLILHTIGSHGPTYYERYTEAERKFTP

TCDTNEIDKCARATLVNTYDNTVLYVDQFIDKVIRKLENRDDLESAVYYVSDHGESLGENGMYLHAAPYAIAPSGQTHIP

MVMWFSKAFRQHGGIDFQCLKQKAAENEYSHDHYFSTVLGLMDISNSQTYRKEMDILAACRRPR

>Neisseria bergeri_ LptA

MIKPNLRPKLGSSVLIAFLSLYSSLVLNYAFFSKVVELRPFNGTGADIFLYTMPVVLFFLSNFVFHVIALPFVHKVLIPL

ILVISAAVSYQEIFFNIYFNKSMLNNVLQTTAAESARLITPGYVLWIVCLGVLPALAYIAVKVKYRVWYKELLTRLVLAA

VSFLCALGIAMLQYQDYASFFRNNKSVTHLIVPSNFIGAGVSKYKDWKRSNIPYTQLDMAVVQNRPVGSLRRFVVLVVGE

TTRAANWGLNGYSRQTTPLLAARGDEIVNFPQVRSCGTSTAHSLPCMFSTFDRTDYDEIKAEHQDNLLDIVQRAGVEVTW

LENDSGCKGVCGKVPNTDVTSLNLPEYCRNGECLDNILLTKFDEALNKNDKDAVLILHTIGSHGPTYYERYTEAERKFTP

TCDTNEIDKCARATLVNTYDNTVLYVDQFIDKVIRKLENRDDLESVVHYVSDHGESLGENGMYLHAAPYAIAPSGQTHIP

MVMWFSKAFRQHGGIDFQCLKQKAAENEYSHDHYFSTVLGLMDISNSQTYRKEMDILAACRRPR

>*Neisseria polysaccharea*_ LptA

MIKPNLRPKLGSSVLIAFLSLYSSLVLNYAFFTKVVELHPFNGTGADIFLYTMPVVLFFLSNFVFHVIALPFVHKVLIPL

ILVISAAVSYQEIFFNIYFNKSMLNNVLQTTAAESARLITPGYVLWIVCLGVLPALAYIAVKVKYRVWYKELLTRLVLAA

VSFLCALGIAMLQYQDYASFFRNNKSVTHLIVPSNFIGAGVSKYKDWKRSNIPYTQLDMAVVQNRPAGSLRRFVVLIVGE

TTRAANWGLNGYSRQTTPLLAARGDEIVNFPQVRSCGTSTAHSLPCMFSTFDRTDYDEIKAEHQDNLLDIVQRAGVEVTW

LENDSGCKGVCGKVPNTDVTSLNLPEYCRNGECLDNILLTKFDEALNKNDKDAVLILHTIGSHGPTYYERYTEAERKFTP

TCDTNEIDKCARATLVNTYDNTVLYVDQFIDKVIRKLENRDDLESAVHYVSDHGESLGENGMYLHAAPYAIAPSGQTHIP

MVMWFSKAFRQHGGIDFQCLKQKAAENEYSHDHYFSTVLGLMDISNSQTYRKDMDILAACRRPR

>*Neisseria lactamica*_ LptA

MIKPNLRPKLGSSALIAFLSLYFSLVLNYAFFAKVVELHPFNGTGADIFLYTMPVVLFFLSNFVFHAIALPFVHKVLIPL

ILVISAAVSYQEIFFNIYFNKSMLNNVLQTTAAESARLITPGYVLWIVCLGVLPALAYIAVKVKYRVWYKELLTRLVLAA

VSFLCALGIAMLQYQDYASFFRNNKSVTHLIVPSNFIGAGVSKYKDWKRSNIPYTQLDMAVVQNRPAGSLRRFVVLIVGE

TTRAANWGLNGYSRQTTPLLAARGDEIVNFPQVRSCGTSTAHSLPCMFSTFDRTDYDEIKAEHQDNLLDIVQRAGVEVTW

LENDSGCKGVCGKVPNTDVTSLNLPEYCRNGECLDNILLTKFDEALNKNDKDAVLILHTIGSHGPTYYERYTEAERKFTP

TCDTNEIDKCARATLVNTYDNTVLYVDQFIDKVIRKLENRDDLESAVHYVSDHGESLGENGMYLHAAPYAIAPSGQTHIP

MVMWFSKAFRQHGGIDFQCLKQKAAENEYSHDHYFSTVLGLMDISNSQTYRKDMDILAACRRPR

>*Neisseria meningitides*_LptA

MIKPNLRPKLGSSALIAFLSLYSSLVLNYAFFAKVVELHPFNGTGADIFLYTMPVVLFFLSNFVFHVIALPFVHKVLIPL

ILVISAAVSYQEIFFNIYFNKSMLNNVLQTTAAESARLITPGYVLWIVCLGVLPALAYIAVKVKYRVWYKELLTRLVLAA

VSFLCALGIAMLQYQDYASFFRNNKSVTHLIVPSNFIGAGVSKYKDWKRSNIPYTQLDMAVVQNRPTGSLRRFVVLVVGE

TTRAANWGLNGYSRQTTPLLAARGDEIVNFPQVRSCGTSTAHSLPCMFSTFDRTDYDEIKAEHQDNLLDIVQRAGVEVTW

LENDSGCKGVCGKVPNTDVTSLNLPEYCRNGECLDNILLTKFDEVLNKNDKDAVLILHTIGSHGPTYYERYTEAERKFTP

TCDTNEINKCTRATLVNTYDNTVLYVDQFIDKVIRKLENRDDLESVVHYVSDHGESLGENGMYLHAAPYAIAPSGQTHIP

MVMWFSKAFRQHGGIDFQCLKQKAAENEYSHDHYFSTVLGLMDISNSQTYRKEMDILAACRRPR

>*Proteobacteria*_ LptA

MIKPNLRPKLGSSALIAFLSLYSSLVLNYAFFAKVVELHPFNGTGADIFLYTMPVVLFFLSNFVFHVIALPFVHKVLIPL

ILVISAAVSYQEIFFNIYFNKSMLNNVLQTTAAESARLITPGYVLWIVCLGVLPALAYIAVKVKYRVWYKELLTRLVLAA

VSFLCALGIAMLQYQDYASFFRNNKSVTHLIVPSNFIGAGVSKYKDWKRSNIPYTQLDMAVVQNRPVGSLRRFVVLVVGE

TTRAANWGLNGYSRQTTPLLAARGDEIVNFPQVRSCGTSTAHSLPCMFSTFDRTDYDEIKAEHQDNLLDIVQRAGVEVTW

LENDSGCKGVCGKVPNTDVTSLNLPEYCRNGECLDNILLTKFDEVLNKNDKDAVLILHTIGSHGPTYYERYTEAERKFTP

TCDTNEINKCTRATLVNTYDNTVLYVDQFIDKVIRKLENRDDLESVVHYVSDHGESLGENGMYLHAAPYAIAPSGQTHIP

MVMWFSKAFRQHGGIDFQCLKQKAAENEYSHDHYFSTVLGLMDISNSQTYRKEMDILAACRRPR

>*Lautropia* sp._ LptA

MRLNRLHASSATLIAVVSLYFTVAFNIAFYKKVVELNGGADWFVYSLPVLLFFLLNGVFQLLAAPLLHKVVIPALLVIGA

AISYQSLFFNIYFDKHMLTNVLITGWAESSRLMTPAYLGWIAGLGVVPALLYLMVKVDYRRWYKEIAQRAVLIVLSVLVI

GGVARYFYQDYASFFRNNRDVTHLIVPTNLIVAGGSKLKTWHRARMPYMQLDLAVTQAKPDNHRHFVVLIVGETTRAQNW

GLNGYARQTTPRLAKRGDEVINFHDVSSCGTSTAHSVPCMFSNMNRVGYNDARAARQDNLLDILQRAGVDISWLDNDTGC

KGVCKNVAATVDLTALNLPEFCRNGECLDDILLPAFDEILNKESGKDTLVVLHTMGSHGPTYYERYTAEDRTFTPTCDTN

EINRCSKEQLVNTYDNTIVYVDRFIDRVIGRLEKRDDLESAVLYVSDHGESLGENGVYLHGTPYGIATKEQTQVPMIMWF

SKAFRQNEGVDFQCLADNARKNTYSHDNYFSTVFGMMDMALALSETYRQDMDILAPCRTPRPQATGKDARADRHPAAQR

>*Lautropia mirabilis*_ LptA

MYSLPVLLFFLLNGVFQLLAAPLLHKVVIPALLVIGAAISYQSLFFNIYFDKHMLTNVLITGWAESSRLMTPAYLGWIAG

LGVVPALLYLMVKVDYRRWYKEIAQRAVLIVLSVLVIGGVARYFYQDYASFFRNNRDVTHLIVPTNLIVAGGSKLKTWHR

ARMPYMQLDLAVTQAKPDNHRHFVVLIVGETTRAQNWGLNGYARQTTPRLAKRGDEVINFHDVSSCGTSTAHSVPCMFSN

MNRVGYNDARAARQDNLLDILQRAGVDISWLDNDTGCKGVCKNVAATVDLTALNLPEFCRNGECLDDILLPAFDEILNKE

SGKDTLVVLHTMGSHGPTYYERYTAEDRTFTPTCDTNEINKCSKEQLVNTYDNTIVYVDRFIDRVIGRLEKRDDLESAVL

YVSDHGESLGENGVYLHGTPYGIATKEQTQVPMVMWFSKAFRQNEGVDFQCLADHARKNTYSHDHYFSTVFGMMDMALAL

SETYRQDMDILAPCRTPRPQVPGKDARADRHPAAQR

>*Gallibacterium anatis*_ LptA

MQKIALSMRSSTLMALVALYFTLILNYAFYAKVLSIHPFTGAAGDYFLLTVPFFVFFVLNAVFQILALPLLHKVLIPALL

IISAAIGYSEVFLDVYFTTDMLENVLQTNFAESSRMMTLPYIAWIIGFGVVPALLYLLVKVDYRVWYKEIAYRIGLILLS

VVVIVGIAKVFYQDYAGFVRNNKSVPALIVPSNFISAGINEIKRIREANMPYTQLGLDAAQEKPDDYRHFTVLVVGETTR

AQNWGLNGYQPQTTPLLAARGKQIINFRNVSSCGTATALSVPCMFSGMGRDGYDAVKASHQDNILDVLQRAGVEVIWLDN

DSGCKGVCERVLNKDITATNDPQYCKDGECLDNILLSYIDDVIKKTHKDTVLVLHTIGSHGPTYYERYSEQYRRFTPTCD

TNEIQKCSNEQLVNTYNNGILYIDQFLDKVIAKVEAHPELEAAVYYLSDHGESLGENGVYLHGTPYAIAPEQQTRIPMIM

WFSDRFRQNENIDFDCLERNAQQQSYSHDNLYSTLFGLMDMNPNTSVYKKELDIITQCKKP
