# Supplementary material for: Multidrug Antimicrobial Resistance and Molecular Detection of mcr-1 Gene in Salmonella Species Isolated from Chicken
Source: Animals (Basel). 2021 Jan 15;11(1):206. doi: 10.3390/ani11010206 (PMC7829884; doi:10.3390/ani11010206)
Supplement: Supplementary file 1 [file animals-11-00206-s001.zip › Supplementary File S3_14012021.docx]

**Supplementary File S3**

**The multiple sequence alignments of SAUVM MCR-1 proteins with putative conserved sites of other *Salmonella* MCR-1.**

SAUVM_S6         MMQHTSVWYRRSVSPFVLVASVAVFLTATANLTFFDKISQTYPIADNLGFVLTIAVVLFGAMLLIT
SAUVM_S7         MMQHTSVWYRRSVSPFVLVASVAVFLTATANLTFFDKISQTYPIADNLGFVLTIAVVLFGAMLLIT
SAUVM_S8         MMQHTSVWYRRSVSPFVLVASVAVFLTATANLTFFDKISQTYPIADNLGFVLTIAVVLFGAMLLIT
SAUVM_S9         MMQHTSVWYRRSVIPFVLVASVAVFLTATANLTFFDKISQTYPIADNLGFVMTIAVLLFGPMLLIT
SAUVM_S10        MMQHTSVWYRRSVSPFVLVASVAVFLTATANLTFFDKISQTYPIADNLGFVLTIAVVLFGAMLLIT
WP_129336087_S   MMQHTSVWYRRSVSPFVLVASVAVFLTATANLTFFDKISQTYPIADNLGFVLTIAVVLFGAMLLIT
EAN4289655_S     MMQHTSVWYRRSVSPFVLVASVAVFLTATANLTFFDKISQTYPIADNLGFVLTIAVVLFGAMLLIT
ECM8937208_S     MMQHTSVWYRRSVSPFVLVARVAVFLTATANLTFFDKISQTYPIADNLGFVLTIAVVLFGAMLLIT
EBR5928579_S     MMQHTSVWYRRSVSPFVLVASVAVFLTATANLTFFDKISQTYPIADNLGFVLTIAVVLFGAMLLIT
APY22148_S       -MQHTSVWYRRSVSPFVLVASVAVFLTATANLTFFDKISQTYPIADNLGFVLTIAVVLFGAMLLIT
WP_077248208_S   MMQHTSVWYRRSVSPFVLVASVAVFLTATANLTFFDKISQTYPIADNLGFVLTIAVVLFGAMLLIT
WP_127821221_S   MMQHTSVWYRRSVSPFVLVASVAVFLTATANLTFFDKISQTYPIADNLGFVLTIAVVLFGAMLLIT
ATG71348_S       -----------------LVASVAVFLTATANLTFFDKISQTYPIADNLGFVLTIAVVLFGAMLLIT
                                  *** ******************************:****:***.*****


SAUVM_S6         TLLSSYRYVLKPVLILLLIMGAVTSYFTDTYGTVYDTTMLQNALQTDQAETKDLLNAAFIMRIIGL
SAUVM_S7         TLLSSYRYVLKPVLILLLIMGAVTSYFTDTYGTVYDTTMLQNALQTDQAETKDLLNAAFIMRIIGL
SAUVM_S8         TLLSSYRYVLKPVLILLLIMGAVTSYFTDTYGTVYDTTMLQNALQTDQAETKDLLNAAFIMRIIGL
SAUVM_S9         TMLSSHRYVLKTVLILLLIMDAVTNYFTDTYGTVYDTTMLQNALQTDQAETKDLLNAAFIMRIIGL
SAUVM_S10        TLLSSYRYVLKPVLILLLIMGAVTSYFTDTYGTVYDTTMLQNALQTDQAETKDLLNAAFIMRIIGL
WP_129336087_S   TLLSSYRYVLKPVLILLLIMGAVTSYFTDTYGTVYDTTMLQNALQTDQAETKDLLNAAFIMRIIGL
EAN4289655_S     TLLSSYRYVLKPVLILLLIMGAVTSYFTDTYGTVYDTTMLQNALQTDQAETKDLLNAAFIMRIIGL
ECM8937208_S     TLLSSYRYVLKPVLILLLIMGAVTSYFTDTYGTVYDTTMLQNALQTDQAETKDLLNAAFIMRIIGL
EBR5928579_S     TLLSSYRYVLKPVLILLLIMGAVTSYFTDTYGTVYDTTMLQNALQTDQAETKDLLNAAFIMRIIGL
APY22148_S       TLLSSYRYVLKPVLILLLIMGAVTSYFTDTYGTVYDTTMLQNALQTDQAETKDLLNAAFIMRIIGL
WP_077248208_S   TLLSSYRYVLKPVLILLLIMGAVTSYFTDTYGTVYDTTMLQNALQTDQAETKDLLNAAFIMRIIGL
WP_127821221_S   TLLSSYRYVLKPVLILLLIMGAVTSYFTDTYGTVYDTTMLQNALQTDQAETKDLLNAAFIMRIIGL
ATG71348_S       TLLSSYRYVLKPVLILLLIMGAVTSYFTDTYGTVYDTTMLQNALQTDQAETKDLLNAAFIMRIIGL
                 *:***:*****.********.***.*****************************************


SAUVM_S6         GVLPSLLVAFVKVDYPTWGKGLMRRLGLIVASLALILLPVVAFSSHYASFFRVHKPLRSYVNPIMP
SAUVM_S7         GVLPSLLVAFVKVDYPTWGKGLMRRLGLIVASLALILLPVVAFSSHYASFFRVHKPLRSYVNPIMP
SAUVM_S8         GVLPSLLVAFVKVDYPTWGKGLMRRLGLIVASLALILLPVVAFSSHYASFFRVHKPLRSYVNPIMP
SAUVM_S9         GVLPSLXVAFVKVDYPTWGKGLMRRLGLIVASLALILLPVVAFSSHYASFFRVHKPLRSYVNPIMP
SAUVM_S10        GVLPSLLVAFVKVDYPTWGKGLMRRLGLIVASLALILLPVVAFSSHYASFFRVHKPLRSYVNPIMP
WP_129336087_S   GVLPSLLVAFVKVDYPTWGKGLMRRLGLIVASLALILLPVVAFSSHYASFFRVHKPLRSYVNPIMP
EAN4289655_S     GVLPSLLVAFVKVDYPTWGKGLMRRLGLIVASLALILLPVVAFSSHYASFFRVHKPLRSYVNPIMP
ECM8937208_S     GVLPSLLVAFVKVDYPTWGKGLMRRLGLIVASLALILLPVVAFSSHYASFFRVHKPLRSYVNPIMP
EBR5928579_S     GVLPSLLVAFVKVDYPTWGKGLMRRLGLIVASLALILLPVVAFSSHYASFFRVHKPLRRYVNPIMP
APY22148_S       GVLPSLLVAFVKVDYPTWGKGLMRRLGLIVASLALILLPVVAFSSHYASFFRVHKPLRSYVNPIMP
WP_077248208_S   GVLPSLLVAFVKVDYPTWGKGLMRRLGLIVASLALILLPVVAFSSHYASFFRVHKPLRSYVNPIMP
WP_127821221_S   GVLPSLLVAFVKVDYPTWGKGLMRRLGLIVASLALILLPVVAFSSHYASFFRVHKPLRSYVNPIMP
ATG71348_S       GVLPSLLVAFVKVDYPTWGKGLMRRLGLIVASLALILLPVVAFSSHYASFFRVHKPLRSYVNPIMP
                 ****** *************************************************** *******


SAUVM_S6         IYSVGKLASIEYKKASAPKDTIYHAKDAVQATKPDMRKPRLVVFVVGETARADHVSFNGYERDTFP
SAUVM_S7         IYSVGKLASIEYKKASAPKDTIYHAKDAVQATKPDMRKPRLVVFVVGETARADHVSFNGYERDTFP
SAUVM_S8         IYSVGKLASIEYKKASAPKDTIYHAKDAVQATKPDMRKPRLVVFVVGETARADHVSFNGYERDTFP
SAUVM_S9         IYSVGKLASIEYKKASAPKDTIYHAKDAVQATKPDMRKPRLVVFVVGETARADHVSFNGYERDTFP
SAUVM_S10        IYSVGKLASIEYKKASAPKDTIYHAKDAVQATKPDMRKPRLVVFVVGETARADHVSFNGYERDTFP
WP_129336087_S   IYSVGKLASIEYKKASAPKDTIYHAKDAVQATKPDMRKPRLVVFVVGETARADHVSFNGYERDTFP
EAN4289655_S     IYSVGKLASIEYKKASAPKDTIYHAKDAVQATKPDMRKPRLVVFVVGETARADHVSFNGYERDTFP
ECM8937208_S     IYSVGKLASIEYKKASAPKDTIYHAKDAVQATKPDMRKPRLVVFVVGETARADHVSFNGYERDTFP
EBR5928579_S     IYSVGKLASIEYKKASAPKDTIYHAKDAVQATKPDMRKPRLVVFVVGETARADHVSFNGYERDTFP
APY22148_S       IYSVGKLASIEYKKASAPKDTIYHAKDAVQATKPDMRKPRLVVFVVGETARADHVSFNGYERDTFP
WP_077248208_S   IYSVGKLASIEYKKASAPKDTIYHAKDAVQATKPDMRKPRLVVFVVGETARADHVSFNGYERDTFP
WP_127821221_S   IYSVGKLASIEYKKASAPKDTIYHAKDAVQATKPDMRKPRLVVFVVGETARADHVSFNGYERDTFP
ATG71348_S       IYSVGKLASIEYKKASAPKDTIYHAKDAVQATKPDMRKPRLVVFVVGETARADHVSFNGYERDTFP
                 ******************************************************************


SAUVM_S6         QLAKIDGVTNFSNVTSCGTSTAYSVPCMFSYLGADEYDVDTAKYQENVLDTLDRLGVSILWRDNNS
SAUVM_S7         QLAKIDGVTNFSNVTSCGTSTAYSVPCMFSYLGADEYDVDTAKYQENVLDTLDRLGVSILWRDNNS
SAUVM_S8         QLAKIDGVTNFSNVTSCGTSTAYSVPCMFSYLGADEYDVDTAKYQENVLDTLDRLGVSILWRDNNS
SAUVM_S9         QLAKIDGVTNFSNVTSCGTSTAYSVPCMFSYLGADEYDVDTAKYQENVLDTLDRLGVSILWRDNNS
SAUVM_S10        QLAKIDGVTNFSNVTSCGTSTAYSVPCMFSYLGADEYDVDTAKYQENVLDTLDRLGVSILWRDNNS
WP_129336087_S   QLAKIDGVTNFSNVTSCGTSTAYSVPCMFSYLGADEYDVDTAKYQENVLDTLDRLGVSILWRDNNS
EAN4289655_S     QLAKIDGVTNFSNVTSCGTSTAYSVPCMFSYLGADEYDVDTAKYQENVLDTLDRLGVSILWRDNNS
ECM8937208_S     QLAKIDGVTNFSNVTSCGTSTAYSVPCMFSYLGADEYDVDTAKYQENVLDTLDRLGVSILWRDNNS
EBR5928579_S     QLAKIDGVTNFSNVTSCGTSTAYSVPCMFSYLGADEYDVDTAKYQENVLDTLDRLGVSILWRDNNS
APY22148_S       QLAKIDGVTNFSNVTSCGTSTAYSVPCMFSYLGADEYDVDTAKYQENVLDTLDRLGVSILWRDNNS
WP_077248208_S   QLAKIDGVTNFSNVTSCGTSTAYSVPCMFSYLGADEYDVDTAKYQENVLDTLDRLGVSILWRDNNS
WP_127821221_S   QLAKIDGVTNFSNVTSCGTSTAYSVPCMFSYLGADEYDVDTP-IPRNVLDTLDRLGVSILWRDNNS
ATG71348_S       QLAKIDGVTNFSNVTSCGTSTAYSVPCMFSYLGADEYDVDTAKYQENVLDTLDRLGVSILWRDNNS
                 *****************************************.   .********************


SAUVM_S6         DSKGVMDKLPKAQFADYKSATNNAICNTNPYNECRDVGMLVGLDDFVAANNGKDMLIMLHQMGNHG
SAUVM_S7         DSKGVMDKLPKAQFADYKSATNNAICNTNPYNECRDVGMLVGLDDFVAANNGKDMLIMLHQMGNHG
SAUVM_S8         DSKGVMDKLPKAQFADYKSATNNAICNTNPYNECRDVGMLVGLDDFVAANNGKDMLIMLHQMGNHG
SAUVM_S9         DSKGVMDKLPKAQFADYKSATNNAICNTNPYNECRDVGMLVGLDDFVAANNGKDMLIMLHQMGNHG
SAUVM_S10        DSKGVMDKLPKAQFADYKSATNNAICNTNPYNECRDVGMLVGLDDFVAANNGKDMLIMLHQMGNHG
WP_129336087_S   DSKGVMDKLPKAQFADYKSATNNAICNTNPYNECRDVGMLVGLDDFVAANNGKDMLIMLHQMGNHG
EAN4289655_S     DSKGVMDKLPKAQFADYKSATNNAICNTNPYNECRDVGMLVGLDDFVAANNGKDMLIMLHQMGNHG
ECM8937208_S     DSKGVMDKLPKAQFADYKSATNNAICNTNPYNECRDVGMLVGLDDFVAANNGKDMLIMLHQMGNHG
EBR5928579_S     DSKGVMDKLPKAQFADYKSATNNAICNTNPYNECRDVGMLVGLDDFVAANNGKDMLIMLHQMGNHG
APY22148_S       DSKGVMDKLPKAQFADYKSATNNAICNTNPYNECRDVGMLVGLDDFVAANNGKDMLIMLHQMGNHG
WP_077248208_S   DSKGVMDKLPKAQFADYKSATNNAICNTNPYNECRDVGMLVGLDDFVAANNGKDMLIMLHQMGNHG
WP_127821221_S   DSKGVMDKLPKAQFADYKSATNNAICNTNPYNECRDVGMLVGLDDFVAANNGKDMLIMLHQMGNHG
ATG71348_S       DSKGVMDKLPKAQFADYKSATNNAICNTNPYNECRDVGMLVGLDDFVAANNGKDMLIMLHQMGNHG
                 ******************************************************************


SAUVM_S6         PAYFKRYDEKFAKFTPVCEGNELAKCEHQSLINAYDNALLATDDFIAQSIQWLQTHSNAYDVSMLY
SAUVM_S7         PAYFKRYDEKFAKFTPVCEGNELAKCEHQSLINAYDNALLATDDFIAQSIQWLQTHSNAYDVSMLY
SAUVM_S8         PAYFKRYDEKFAKFTPVCEGNELAKCEHQSLINAYDNALLATDDFIAQSIQWLQTHSNAYDVSMLY
SAUVM_S9         PAYFKRYDEKFAKFTPVCEGNELAKCEHQSLINAYDNALLATDDFIAQSIQWLQTHSNAYDVSMLY
SAUVM_S10        PAYFKRYDEKFAKFTPVCEGNELAKCEHQSLINAYDNALLATDDFIAQSIQWLQTHSNAYDVSMLY
WP_129336087_S   PAYFKRYDEKFAKFTPVCEGNELAKCEHQSLINAYDNALLATDDFIAQSIQWLQTHSNAYDVSMLY
EAN4289655_S     PAYFKRYDEKFAKFTPVCEGNELAKCEHQSLINAYDNALLATDDFIAQSIQWLQTHSNAYDVSMLY
ECM8937208_S     PAYFKRYDEKFAKFTPVCEGNELAKCEHQSLINAYDNALLATDDFIAQSIQWLQTHSNAYDVSMLY
EBR5928579_S     PAYFKRYDEKFAKFTPVCEGNELAKCEHQSLINAYDNALLATDDFIAQSIQWLQTHSNAYDVSMLY
APY22148_S       PAYFKRYDEKFAKFTPVCEGNELAKCEHQSLINAYDNALLATDDFIAQSIQWLQTHSNAYDVSMLY
WP_077248208_S   PAYFKRYDEKFAKFTPVCEGNELAKCEHQSLINAYDNALLATDDFIAQSIQWLQTHSNAYDVSMLY
WP_127821221_S   PAYFKRYDEKFAKFTPVCEGNELAKCEHQSLINAYDNALLATDDFIAQSIQWLQTHSNAYDVSMLY
ATG71348_S       PAYFKRYDEKFAKFTPVCEGNELAKCEHQSLINAYDNALLATDDFIAQSIQWLQTHSNAYDVSMLY
                 ******************************************************************


SAUVM_S6         VSDHGESLGENGVYLHGMPNAFAPKEQRSVPAFFWTDKQTGITPMATDTVLTHDAITPTLLKLFDV
SAUVM_S7         VSDHGESLGENGVYLHGMPNAFAPKEQRSVPAFFWTDKQTGITPMATDTVLTHDAITPTLLKLFDV
SAUVM_S8         VSDHGESLGENGVYLHGMPNAFAPKEQRSVPAFFWTDKQTGITPMATDTVLTHDAITPTLLKLFDV
SAUVM_S9         VSDHGESLGENGVYLHGMPNAFAPKEQRSVPAFFWTDKQTGITPMATDTVLTHDAITPTLLKLFDV
SAUVM_S10        VSDHGESLGENGVYLHGMPNAFAPKEQRSVPAFFWTDKQTGITPMATDTVLTHDAITPTLLKLFDV
WP_129336087_S   VSDHGESLGENGVYLHGMPNAFAPKEQRSVPAFFWTDKQTGITPMATDTILTHDAITPTLLKLFDV
EAN4289655_S     VSDHGESLGENGVYLHGMPNAFAPQEQRSVPAFFWTDKQTGITPMATDTVLTHDAITPTLLKLFDV
ECM8937208_S     VSDHGESLGENGVYLHGMPNAFAPKEQRSVPAFFWTDKQTGITPMATDTVLTHDAITPTLLKLFDV
EBR5928579_S     VSDHGESLGENGVYLHGMPNAFAPKEQRSVPAFFWTDKQTGITPMATDTVLTHDAITPTLLKLFDV
APY22148_S       VSDHGESLGENGVYLHGMPNAFAPKEQRSVPAFFWTDKQTGITPMATDTVLTHDAITPTLLKLFDV
WP_077248208_S   VSDHGESLGENGVYLHGMPNAFAPKEQRSVPAFFWTDKQTGITPMATDTVLTHDAITPTLLKLFDV
WP_127821221_S   VSDHGESLGENGVYLHGMPNAFAPKEQRSVPAFFWTDKQTGITPMATDTVLTHDAITPTLLKLFDV
ATG71348_S       VSDHGESLGENGVYLHGMPNAFAPKEQRSVPAFFWTDKQTGITPMATDTVLTHDAITPTL------
                 ************************:************************:**********      


SAUVM_S6         TADKVKDR------------------------------TAFIR
SAUVM_S7         TADKVKDR------------------------------TAFIR
SAUVM_S8         TADKVKDR------------------------------TAFIR
SAUVM_S9         TADKVKDR------------------------------TAFIR
SAUVM_S10        TADKVKDR------------------------------TAFIR
WP_129336087_S   TADKVKDR------------------------------TAFIR
EAN4289655_S     TADKVKDR------------------------------TAFIR
ECM8937208_S     TADKVKDR------------------------------TAFIR
EBR5928579_S     TADKVKDR------------------------------TAFIR
APY22148_S       TADKVKDR------------------------------TAFIR
WP_077248208_S   TADKVKDH------------------------------TAFIR
WP_127821221_S   TRTKSKTAPHSSADFSLYFFQTHRTLHSYYGRWGGVCYAVFIK
ATG71348_S       -------------------------------------------
