# Supplementary material for: Multidrug Antimicrobial Resistance and Molecular Detection of mcr-1 Gene in Salmonella Species Isolated from Chicken
Source: Animals (Basel). 2021 Jan 15;11(1):206. doi: 10.3390/ani11010206 (PMC7829884; doi:10.3390/ani11010206)
Supplement: Supplementary file 1 [file animals-11-00206-s001.zip › Supplementary File S4_14012021.docx]

**Supplementary file S4:**

**Alignment of colistin resistance gene *mcr-1* sequences of positive *Salmonella* isolates in the present study.**

SAUVM_S6 ATGATGCAGCATACTTCTGTGTGGTACCGACGCTCGGTCAGTCCGTTTGTTCTTGTGGCG 60

SAUVM_S7 ATGATGCAGCATACTTCTGTGTGGTACCGACGCTCGGTCAGTCCGTTTGTTCTTGTGGCG 60

SAUVM_S8 ATGATGCAGCATACTTCTGTGTGGTACCGACGCTCGGTCAGTCCGTTTGTTCTTGTGGCG 60

SAUVM_S10 ATGATGCAGCATACTTCTGTGTGGTACCGACGCTCGGTCAGTCCGTTTGTTCTTGTGGCG 60

SAUVM_S9 ATGATGCAGCATACTTCTGTGTGGTACCGACGCTCGGTCATTCCGTTTGTTCTTGTGGCG 60

************************************************************

SAUVM_S6 AGTGTTGCCGTTTTCTTGACCGCGACCGCCAATCTTACCTTTTTTGATAAAATCAGCCAA 120

SAUVM_S7 AGTGTTGCCGTTTTCTTGACCGCGACCGCCAATCTTACCTTTTTTGATAAAATCAGCCAA 120

SAUVM_S8 AGTGTTGCCGTTTTCTTGACCGCGACCGCCAATCTTACCTTTTTTGATAAAATCAGCCAA 120

SAUVM_S10 AGTGTTGCCGTTTTCTTGACCGCGACCGCCAATCTTACCTTTTTTGATAAAATCAGCCAA 120

SAUVM_S9 AGTGTTGCCGTTTTCTTGACCGCGACCGCCAATCTTACCTTTTTTGATAAAATCAGCCAA 120

************************************************************

SAUVM_S6 ACCTATCCCATCGCGGACAATCTCGGCTTTGTGCTGACGATCGCTGTCGTGCTCTTTGGC 180

SAUVM_S7 ACCTATCCCATCGCGGACAATCTCGGCTTTGTGCTGACGATCGCTGTCGTGCTCTTTGGC 180

SAUVM_S8 ACCTATCCCATCGCGGACAATCTCGGCTTTGTGCTGACGATCGCTGTCGTGCTCTTTGGC 180

SAUVM_S10 ACCTATCCCATCGCGGACAATCTCGGCTTTGTGCTGACGATCGCTGTCGTGCTCTTTGGC 180

SAUVM_S9 ACCTATCCCATCGCGGACAATCTCGGCTTTGTGATGACAATCGCTGTCTTGCTCTTTGGT 180

************************************************************

SAUVM_S6 GCGATGCTACTGATCACCACGCTGTTATCATCGTATCGCTATGTGCTAAAGCCTGTGTTG 240

SAUVM_S7 GCGATGCTACTGATCACCACGCTGTTATCATCGTATCGCTATGTGCTAAAGCCTGTGTTG 240

SAUVM_S8 GCGATGCTACTGATCACCACGCTGTTATCATCGTATCGCTATGTGCTAAAGCCTGTGTTG 240

SAUVM_S10 GCGATGCTACTGATCACCACGCTGTTATCATCGTATCGCTATGTGCTAAAGCCTGTGTTG 240

SAUVM_S9 CCGATGCTACTGATCACCACGATGTTATCATCGCATCGCTATGTGCTAAAGACTGTGTTG 240

***********************************************************

SAUVM_S6 ATTTTGCTATTAATCATGGGCGCGGTGACCAGTTATTTTACTGACACTTATGGCACGGTC 300

SAUVM_S7 ATTTTGCTATTAATCATGGGCGCGGTGACCAGTTATTTTACTGACACTTATGGCACGGTC 300

SAUVM_S8 ATTTTGCTATTAATCATGGGCGCGGTGACCAGTTATTTTACTGACACTTATGGCACGGTC 300

SAUVM_S10 ATTTTGCTATTAATCATGGGCGCGGTGACCAGTTATTTTACTGACACTTATGGCACGGTC 300

SAUVM_S9 ATTTTGCTCTTAATCATGGACGCGGTGACCAATTATTTTACTGACACTTATGGCACGGTC 300

************************************************************

SAUVM_S6 TATGATACGACCATGCTCCAAAATGCCCTACAGACCGACCAAGCCGAGACCAAGGATCTA 360

SAUVM_S7 TATGATACGACCATGCTCCAAAATGCCCTACAGACCGACCAAGCCGAGACCAAGGATCTA 360

SAUVM_S8 TATGATACGACCATGCTCCAAAATGCCCTACAGACCGACCAAGCCGAGACCAAGGATCTA 360

SAUVM_S10 TATGATACGACCATGCTCCAAAATGCCCTACAGACCGACCAAGCCGAGACCAAGGATCTA 360

SAUVM_S9 TATGATACGACCATGCTCCAAAATGCCCTACAGACCGACCAAGCCGAGACCAAGGATCTA 360

************************************************************

SAUVM_S6 TTAAACGCAGCGTTTATCATGCGTATCATTGGTTTGGGTGTGCTACCAAGTTTGCTTGTG 420

SAUVM_S7 TTAAACGCAGCGTTTATCATGCGTATCATTGGTTTGGGTGTGCTACCAAGTTTGCTTGTG 420

SAUVM_S8 TTAAACGCAGCGTTTATCATGCGTATCATTGGTTTGGGTGTGCTACCAAGTTTGCTTGTG 420

SAUVM_S10 TTAAACGCAGCGTTTATCATGCGTATCATTGGTTTGGGTGTGCTACCAAGTTTGCTTGTG 420

SAUVM_S9 TTAAACGCAGCGTTTATCATGCGTATCATTGGTTTGGGTGTGCTACCAAGTTTGCTNGTG 420

************************************************************

SAUVM_S6 GCTTTTGTTAAGGTGGATTATCCGACTTGGGGCAAGGGTTTGATGCGCCGATTGGGCTTG 480

SAUVM_S7 GCTTTTGTTAAGGTGGATTATCCGACTTGGGGCAAGGGTTTGATGCGCCGATTGGGCTTG 480

SAUVM_S8 GCTTTTGTTAAGGTGGATTATCCGACTTGGGGCAAGGGTTTGATGCGCCGATTGGGCTTG 480

SAUVM_S10 GCTTTTGTTAAGGTGGATTATCCGACTTGGGGCAAGGGTTTGATGCGCCGATTGGGCTTG 480

SAUVM_S9 GCTTTTGTTAAGGTGGATTATCCGACTTGGGGCAAGGGTTTGATGCGCCGATTGGGCTTG 480

************************************************************

SAUVM_S6 ATCGTGGCAAGTCTTGCGCTGATTTTACTGCCTGTGGTGGCGTTCAGCAGTCATTATGCC 540

SAUVM_S7 ATCGTGGCAAGTCTTGCGCTGATTTTACTGCCTGTGGTGGCGTTCAGCAGTCATTATGCC 540

SAUVM_S8 ATCGTGGCAAGTCTTGCGCTGATTTTACTGCCTGTGGTGGCGTTCAGCAGTCATTATGCC 540

SAUVM_S10 ATCGTGGCAAGTCTTGCGCTGATTTTACTGCCTGTGGTGGCGTTCAGCAGTCATTATGCC 540

SAUVM_S9 ATCGTGGCAAGTCTTGCGCTGATTTTACTGCCTGTGGTGGCGTTCAGCAGTCATTATGCC 540

************************************************************

SAUVM_S6 AGTTTCTTTCGCGTGCATAAGCCGCTGCGTAGCTATGTCAATCCGATCATGCCAATCTAC 600

SAUVM_S7 AGTTTCTTTCGCGTGCATAAGCCGCTGCGTAGCTATGTCAATCCGATCATGCCAATCTAC 600

SAUVM_S8 AGTTTCTTTCGCGTGCATAAGCCGCTGCGTAGCTATGTCAATCCGATCATGCCAATCTAC 600

SAUVM_S10 AGTTTCTTTCGCGTGCATAAGCCGCTGCGTAGCTATGTCAATCCGATCATGCCAATCTAC 600

SAUVM_S9 AGTTTCTTTCGCGTGCATAAGCCGCTGCGTAGCTATGTCAATCCGATCATGCCAATCTAC 600

************************************************************

SAUVM_S6 TCGGTGGGTAAGCTTGCCAGTATTGAGTATAAAAAAGCCAGTGCGCCAAAAGATACCATT 660

SAUVM_S7 TCGGTGGGTAAGCTTGCCAGTATTGAGTATAAAAAAGCCAGTGCGCCAAAAGATACCATT 660

SAUVM_S8 TCGGTGGGTAAGCTTGCCAGTATTGAGTATAAAAAAGCCAGTGCGCCAAAAGATACCATT 660

SAUVM_S10 TCGGTGGGTAAGCTTGCCAGTATTGAGTATAAAAAAGCCAGTGCGCCAAAAGATACCATT 660

SAUVM_S9 TCGGTGGGTAAGCTTGCCAGTATTGAGTATAAAAAAGCCAGTGCGCCAAAAGATACCATT 660

************************************************************

SAUVM_S6 TATCACGCCAAAGACGCGGTACAAGCAACCAAGCCTGATATGCGTAAGCCACGCCTAGTG 720

SAUVM_S7 TATCACGCCAAAGACGCGGTACAAGCAACCAAGCCTGATATGCGTAAGCCACGCCTAGTG 720

SAUVM_S8 TATCACGCCAAAGACGCGGTACAAGCAACCAAGCCTGATATGCGTAAGCCACGCCTAGTG 720

SAUVM_S10 TATCACGCCAAAGACGCGGTACAAGCAACCAAGCCTGATATGCGTAAGCCACGCCTAGTG 720

SAUVM_S9 TATCACGCCAAAGACGCGGTACAAGCAACCAAGCCTGATATGCGTAAGCCACGCCTAGTG 720

************************************************************

SAUVM_S6 GTGTTCGTCGTCGGTGAGACGGCACGCGCCGATCATGTCAGCTTCAATGGCTATGAGCGC 780

SAUVM_S7 GTGTTCGTCGTCGGTGAGACGGCACGCGCCGATCATGTCAGCTTCAATGGCTATGAGCGC 780

SAUVM_S8 GTGTTCGTCGTCGGTGAGACGGCACGCGCCGATCATGTCAGCTTCAATGGCTATGAGCGC 780

SAUVM_S10 GTGTTCGTCGTCGGTGAGACGGCACGCGCCGATCATGTCAGCTTCAATGGCTATGAGCGC 780

SAUVM_S9 GTGTTCGTCGTCGGTGAGACGGCACGCGCCGATCATGTCAGCTTCAATGGCTATGAGCGC 780

************************************************************

SAUVM_S6 GATACTTTCCCACAGCTTGCCAAGATCGATGGCGTGACCAATTTTAGCAATGTCACATCG 840

SAUVM_S7 GATACTTTCCCACAGCTTGCCAAGATCGATGGCGTGACCAATTTTAGCAATGTCACATCG 840

SAUVM_S8 GATACTTTCCCACAGCTTGCCAAGATCGATGGCGTGACCAATTTTAGCAATGTCACATCG 840

SAUVM_S10 GATACTTTCCCACAGCTTGCCAAGATCGATGGCGTGACCAATTTTAGCAATGTCACATCG 840

SAUVM_S9 GATACTTTCCCACAGCTTGCCAAGATCGATGGCGTGACCAATTTTAGCAATGTCACATCG 840

************************************************************

SAUVM_S6 TGCGGCACATCGACGGCGTATTCTGTGCCGTGTATGTTCAGCTATCTGGGCGCGGATGAG 900

SAUVM_S7 TGCGGCACATCGACGGCGTATTCTGTGCCGTGTATGTTCAGCTATCTGGGCGCGGATGAG 900

SAUVM_S8 TGCGGCACATCGACGGCGTATTCTGTGCCGTGTATGTTCAGCTATCTGGGCGCGGATGAG 900

SAUVM_S10 TGCGGCACATCGACGGCGTATTCTGTGCCGTGTATGTTCAGCTATCTGGGCGCGGATGAG 900

SAUVM_S9 TGCGGCACATCGACGGCGTATTCTGTGCCGTGTATGTTCAGCTATCTGGGCGCGGATGAG 900

************************************************************

SAUVM_S6 TATGATGTCGATACCGCCAAATACCAAGAAAATGTGCTGGATACGCTGGATCGCTTGGGC 960

SAUVM_S7 TATGATGTCGATACCGCCAAATACCAAGAAAATGTGCTGGATACGCTGGATCGCTTGGGC 960

SAUVM_S8 TATGATGTCGATACCGCCAAATACCAAGAAAATGTGCTGGATACGCTGGATCGCTTGGGC 960

SAUVM_S10 TATGATGTCGATACCGCCAAATACCAAGAAAATGTGCTGGATACGCTGGATCGCTTGGGC 960

SAUVM_S9 TATGATGTCGATACCGCCAAATACCAAGAAAATGTGCTGGATACGCTGGATCGCTTGGGC 960

************************************************************

SAUVM_S6 GTAAGTATCTTGTGGCGTGATAATAATTCGGACTCAAAAGGCGTGATGGATAAGCTGCCA 1020

SAUVM_S7 GTAAGTATCTTGTGGCGTGATAATAATTCGGACTCAAAAGGCGTGATGGATAAGCTGCCA 1020

SAUVM_S8 GTAAGTATCTTGTGGCGTGATAATAATTCGGACTCAAAAGGCGTGATGGATAAGCTGCCA 1020

SAUVM_S10 GTAAGTATCTTGTGGCGTGATAATAATTCGGACTCAAAAGGCGTGATGGATAAGCTGCCA 1020

SAUVM_S9 GTAAGTATCTTGTGGCGTGATAATAATTCGGACTCAAAAGGCGTGATGGATAAGCTGCCA 1020

************************************************************

SAUVM_S6 AAAGCGCAATTTGCCGATTATAAATCCGCGACCAACAACGCCATCTGCAACACCAATCCT 1080

SAUVM_S7 AAAGCGCAATTTGCCGATTATAAATCCGCGACCAACAACGCCATCTGCAACACCAATCCT 1080

SAUVM_S8 AAAGCGCAATTTGCCGATTATAAATCCGCGACCAACAACGCCATCTGCAACACCAATCCT 1080

SAUVM_S10 AAAGCGCAATTTGCCGATTATAAATCCGCGACCAACAACGCCATCTGCAACACCAATCCT 1080

SAUVM_S9 AAAGCGCAATTTGCCGATTATAAATCCGCGACCAACAACGCCATCTGCAACACCAATCCT 1080

************************************************************

SAUVM_S6 TATAACGAATGCCGCGATGTCGGTATGCTCGTTGGCTTAGATGACTTTGTCGCTGCCAAT 1140

SAUVM_S7 TATAACGAATGCCGCGATGTCGGTATGCTCGTTGGCTTAGATGACTTTGTCGCTGCCAAT 1140

SAUVM_S8 TATAACGAATGCCGCGATGTCGGTATGCTCGTTGGCTTAGATGACTTTGTCGCTGCCAAT 1140

SAUVM_S10 TATAACGAATGCCGCGATGTCGGTATGCTCGTTGGCTTAGATGACTTTGTCGCTGCCAAT 1140

SAUVM_S9 TATAACGAATGCCGCGATGTCGGTATGCTCGTTGGCTTAGATGACTTTGTCGCTGCCAAT 1140

************************************************************

SAUVM_S6 AACGGCAAAGATATGCTGATCATGCTGCACCAAATGGGCAATCACGGGCCTGCGTATTTT 1200

SAUVM_S7 AACGGCAAAGATATGCTGATCATGCTGCACCAAATGGGCAATCACGGGCCTGCGTATTTT 1200

SAUVM_S8 AACGGCAAAGATATGCTGATCATGCTGCACCAAATGGGCAATCACGGGCCTGCGTATTTT 1200

SAUVM_S10 AACGGCAAAGATATGCTGATCATGCTGCACCAAATGGGCAATCACGGGCCTGCGTATTTT 1200

SAUVM_S9 AACGGCAAAGATATGCTGATCATGCTGCACCAAATGGGCAATCACGGGCCTGCGTATTTT 1200

************************************************************

SAUVM_S6 AAGCGATATGATGAAAAGTTTGCCAAATTCACGCCAGTGTGTGAAGGTAATGAGCTTGCC 1260

SAUVM_S7 AAGCGATATGATGAAAAGTTTGCCAAATTCACGCCAGTGTGTGAAGGTAATGAGCTTGCC 1260

SAUVM_S8 AAGCGATATGATGAAAAGTTTGCCAAATTCACGCCAGTGTGTGAAGGTAATGAGCTTGCC 1260

SAUVM_S10 AAGCGATATGATGAAAAGTTTGCCAAATTCACGCCAGTGTGTGAAGGTAATGAGCTTGCC 1260

SAUVM_S9 AAGCGATATGATGAAAAGTTTGCCAAATTCACGCCAGTGTGTGAAGGTAATGAGCTTGCC 1260

************************************************************

SAUVM_S6 AAGTGCGAACATCAGTCCTTGATCAATGCTTATGACAATGCCTTGCTTGCCACCGATGAT 1320

SAUVM_S7 AAGTGCGAACATCAGTCCTTGATCAATGCTTATGACAATGCCTTGCTTGCCACCGATGAT 1320

SAUVM_S8 AAGTGCGAACATCAGTCCTTGATCAATGCTTATGACAATGCCTTGCTTGCCACCGATGAT 1320

SAUVM_S10 AAGTGCGAACATCAGTCCTTGATCAATGCTTATGACAATGCCTTGCTTGCCACCGATGAT 1320

SAUVM_S9 AAGTGCGAACATCAGTCCTTGATCAATGCTTATGACAATGCCTTGCTTGCCACCGATGAT 1320

************************************************************

SAUVM_S6 TTCATCGCTCAAAGTATCCAGTGGCTGCAGACGCACAGCAATGCCTATGATGTCTCAATG 1380

SAUVM_S7 TTCATCGCTCAAAGTATCCAGTGGCTGCAGACGCACAGCAATGCCTATGATGTCTCAATG 1380

SAUVM_S8 TTCATCGCTCAAAGTATCCAGTGGCTGCAGACGCACAGCAATGCCTATGATGTCTCAATG 1380

SAUVM_S10 TTCATCGCTCAAAGTATCCAGTGGCTGCAGACGCACAGCAATGCCTATGATGTCTCAATG 1380

SAUVM_S9 TTCATCGCTCAAAGTATCCAGTGGCTGCAGACGCACAGCAATGCCTATGATGTCTCAATG 1380

************************************************************

SAUVM_S6 CTGTATGTCAGCGATCATGGCGAAAGTCTGGGTGAGAACGGTGTCTATCTACATGGTATG 1440

SAUVM_S7 CTGTATGTCAGCGATCATGGCGAAAGTCTGGGTGAGAACGGTGTCTATCTACATGGTATG 1440

SAUVM_S8 CTGTATGTCAGCGATCATGGCGAAAGTCTGGGTGAGAACGGTGTCTATCTACATGGTATG 1440

SAUVM_S10 CTGTATGTCAGCGATCATGGCGAAAGTCTGGGTGAGAACGGTGTCTATCTACATGGTATG 1440

SAUVM_S9 CTGTATGTCAGCGATCATGGCGAAAGTCTGGGTGAGAACGGTGTCTATCTACATGGTATG 1440

************************************************************

SAUVM_S6 CCAAATGCCTTTGCACCAAAAGAACAGCGCAGTGTGCCTGCATTTTTCTGGACGGATAAG 1500

SAUVM_S7 CCAAATGCCTTTGCACCAAAAGAACAGCGCAGTGTGCCTGCATTTTTCTGGACGGATAAG 1500

SAUVM_S8 CCAAATGCCTTTGCACCAAAAGAACAGCGCAGTGTGCCTGCATTTTTCTGGACGGATAAG 1500

SAUVM_S10 CCAAATGCCTTTGCACCAAAAGAACAGCGCAGTGTGCCTGCATTTTTCTGGACGGATAAG 1500

SAUVM_S9 CCAAATGCCTTTGCACCAAAAGAACAGCGCAGTGTGCCTGCATTTTTCTGGACGGATAAG 1500

************************************************************

SAUVM_S6 CAAACTGGCATCACGCCAATGGCAACCGATACCGTCCTGACCCATGACGCGATCACGCCG 1560

SAUVM_S7 CAAACTGGCATCACGCCAATGGCAACCGATACCGTCCTGACCCATGACGCGATCACGCCG 1560

SAUVM_S8 CAAACTGGCATCACGCCAATGGCAACCGATACCGTCCTGACCCATGACGCGATCACGCCG 1560

SAUVM_S10 CAAACTGGCATCACGCCAATGGCAACCGATACCGTCCTGACCCATGACGCGATCACGCCG 1560

SAUVM_S9 CAAACTGGCATCACGCCAATGGCAACCGATACCGTCCTGACCCATGACGCGATCACGCCG 1560

************************************************************

SAUVM_S6 ACATTATTAAAGCTGTTTGATGTCACCGCGGACAAAGTCAAAGACCGCACCGCATTCATC 1620

SAUVM_S7 ACATTATTAAAGCTGTTTGATGTCACCGCGGACAAAGTCAAAGACCGCACCGCATTCATC 1620

SAUVM_S8 ACATTATTAAAGCTGTTTGATGTCACCGCGGACAAAGTCAAAGACCGCACCGCATTCATC 1620

SAUVM_S10 ACATTATTAAAGCTGTTTGATGTCACCGCGGACAAAGTCAAAGACCGCACCGCATTCATC 1620

SAUVM_S9 ACATTATTAAAGCTGTTTGATGTCACCGCGGACAAAGTCAAAGACCGCACCGCATTCATC 1620

************************************************************

SAUVM_S6 CGCTGA 1626

SAUVM_S7 CGCTGA 1626

SAUVM_S8 CGCTGA 1626

SAUVM_S10 CGCTGA 1626

SAUVM_S9 CGCTGA 1626

******
