# Supplementary material for: Multidrug Antimicrobial Resistance and Molecular Detection of mcr-1 Gene in Salmonella Species Isolated from Chicken
Source: Animals (Basel). 2021 Jan 15;11(1):206. doi: 10.3390/ani11010206 (PMC7829884; doi:10.3390/ani11010206)
Supplement: Supplementary file 1 [file animals-11-00206-s001.zip › Supplementary legends02012021.docx]

**Supplementary Figure S1:** Structure validation by ERRAT (A&B).

**Supplementary Figure S2:** Structure validation by Verify3D server.

**Supplementary Files**

**Supplementary File S1:** Nucleotide sequence of *invA* gene of *Salmonella* isolates SAUVM S6, SAUVM S7, SAUVM S8, SAUVM S9, and to SAUVM S10.

**Supplementary File S2:** Retrieved sequences of MCR-1 and MCR-1 like proteins.

**Supplementary File S3:** The multiple sequence alignments of SAUVM MCR-1 proteins with putative conserved sites of other *Salmonella* MCR-1.

**Supplementary file S4:** Alignment of colistin resistance gene *mcr-1* sequences of positive *Salmonella* isolates in the present study.
