# Supplementary figures and images for: Multidrug Antimicrobial Resistance and Molecular Detection of mcr-1 Gene in Salmonella Species Isolated from Chicken
Source: Animals (Basel). 2021 Jan 15;11(1):206. doi: 10.3390/ani11010206 (PMC7829884; doi:10.3390/ani11010206)

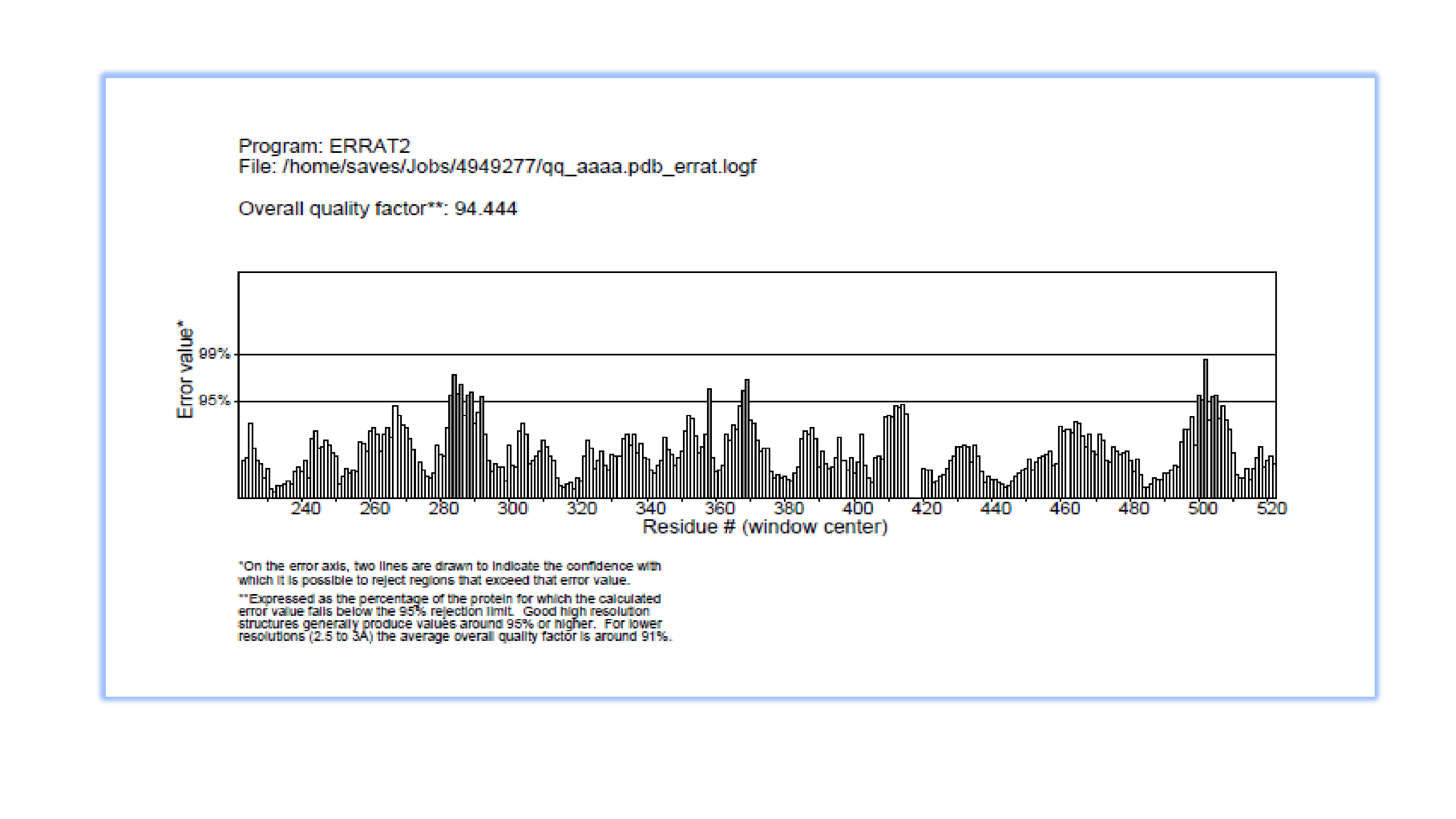

Supplement: Supplementary file 1 [file animals-11-00206-s001.zip › Supplementary-Figure-1A.jpg]

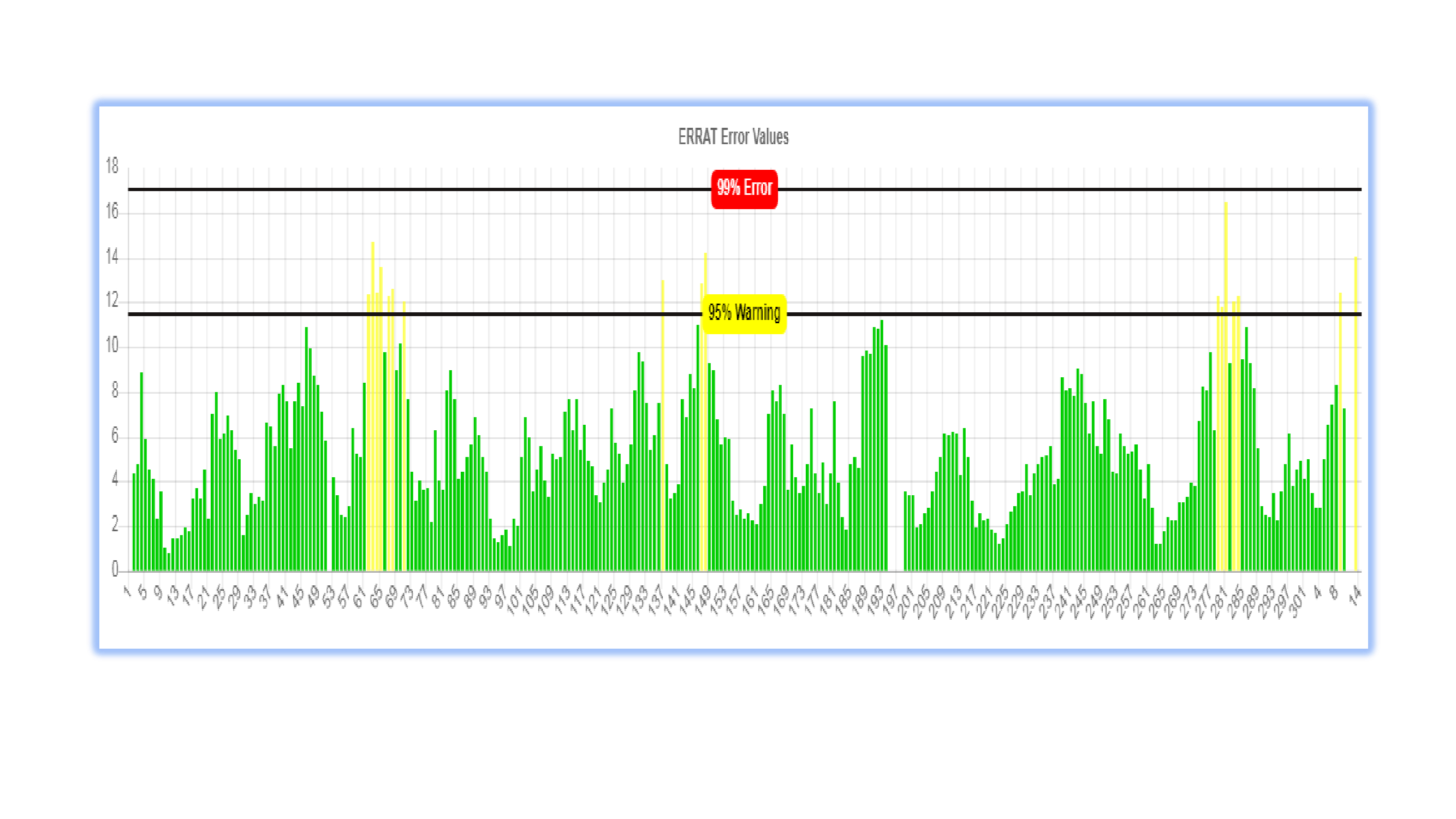

Supplement: Supplementary file 1 [file animals-11-00206-s001.zip › Supplementary-Figure-1B.jpg]

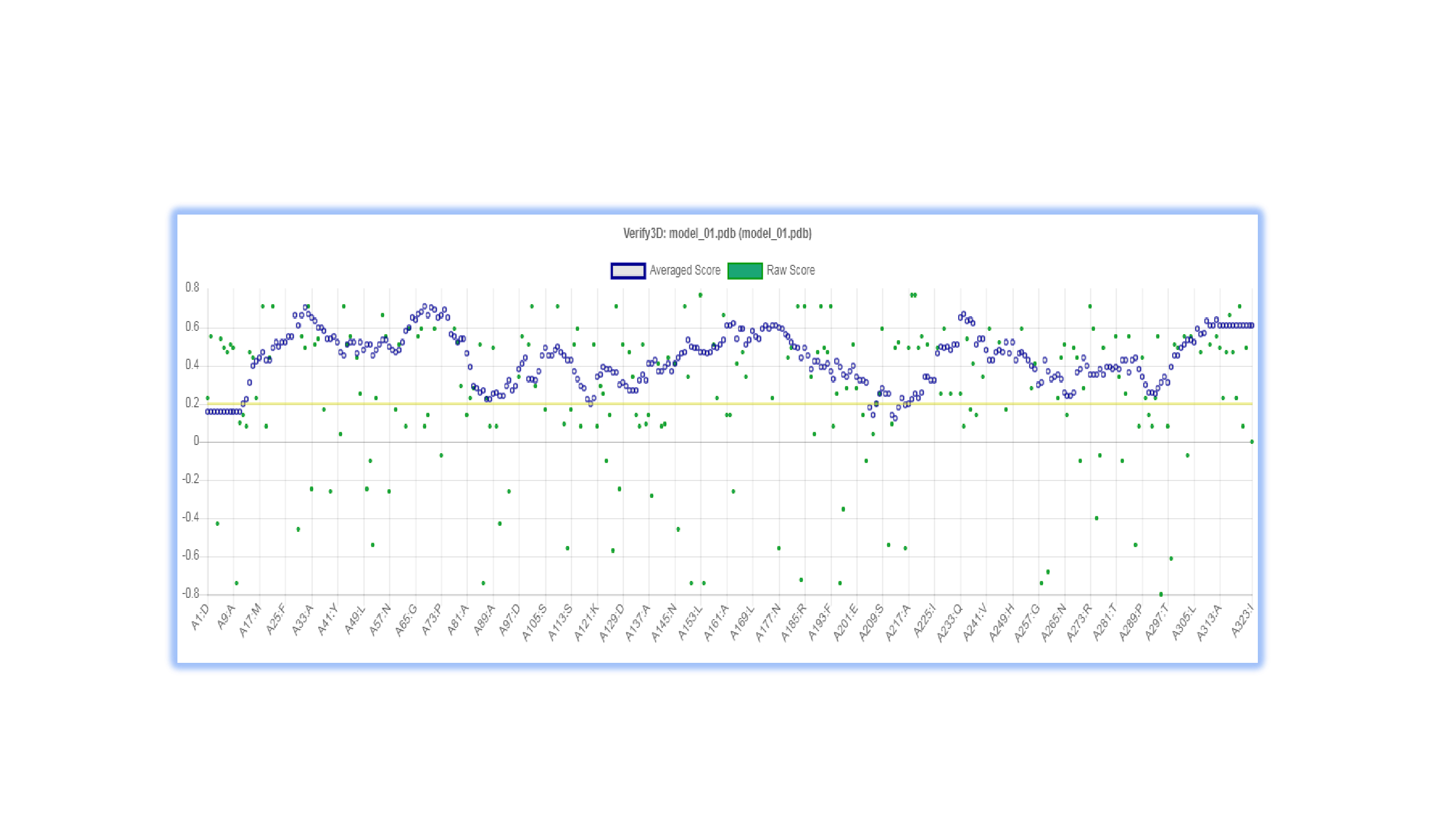

Supplement: Supplementary file 1 [file animals-11-00206-s001.zip › Supplementary-Figure-2.jpg]
